# Research productivity and impact of rehabilitation science faculty across Canada: An observational study of publicly available and survey data

**DOI:** 10.1371/journal.pone.0357806

**Published:** 2026-09-15

**Authors:** Katie L. Kowalski, Joy C. MacDermid, Lochlyn Trefiak, Sophie Albuquerque, Lindsay Moore, Dimitra V. Pouliopoulou, Allyson Page, Christine Meston, Lynn Shaw, Alison Rushton

**Affiliations:** 1 School of Physical Therapy, Western University, London, Ontario, Canada; 2 Roth McFarlane Hand and Upper Limb Centre, St Joseph’s Health Care, London, Ontario, Canada; 3 Lawson Research Institute, St Joseph’s Health Care, London, Ontario, Canada; 4 Department of Surgery, Western University, London, Ontario, Canada; 5 School of Occupational Therapy, Western University, London, Ontario, Canada; 6 Department of Philosophy and Arts, Université du Québec à Trois-Rivières, Trois-Rivières, Quebec, Canada; 7 School of Occupational Therapy, Aix-Marseille Université, Marseille, France; 8 School of Communication Sciences and Disorders, Western University, London, Ontario, Canada; Prince Sattam bin Abdulaziz University, SAUDI ARABIA

## Abstract

**Introduction:**

This study characterized research productivity and impact of rehabilitation science (Physical Therapy, Occupational Therapy, Communication Sciences and Disorders) faculty across Canada.

**Methods:**

Mixed-method cross-sectional design, using publicly available data and online surveys. Faculty were identified from academic unit websites. Bibliometric indices were obtained using Google Scholar and grant funding from Tri-Council databases. Verification protocols checked data validity. Heads of academic units provided research environment data; faculty reported research impact. Descriptive statistics summarized research environment, bibliometric and grant funding indices. Kruskal-Wallis tests compared indices by appointment title. Regression analyses examined associations with h-index and funding; mixed-effects models explored academic unit clustering. Inductive content analysis identified self-described research impact.

**Results:**

Across 41 academic units, 685 full-time faculty were identified. Most units reported research supports, including administrative grant support (pre-award = 97%, post-award = 69%) and research facility access (97%). Median (IQR) h-index was: Full Professors: 32 (21–42), Associate Professors: 19 (14–25), Assistant Professors: 9 (6–16), Full Professors, Teaching: 4 (1–12), Associate Professors, Teaching: 3 (2–7), Assistant Professors, Teaching: 2 (0–5), Instructors: 1 (0–3). Tri-Council funding was concentrated among research-focused appointments and senior ranks. h-index was positively associated with publication count (β = 0.62, *p* < 0.001) and appointment title (*p* < 0.001) but not discipline (*p* = 0.66) or grant funding (*p* = 0.32). h-index variability attributable to academic units was 18.6% (*p* = 0.003), though research environment characteristics were not significant. Tri-Council funding was positively associated with publication count (β = 1.77, *p* < 0.001) and appointment title (*p* = 0.01) but not discipline (*p* = 0.47) or h-index (*p* = 0.32), with minimal between-unit variability (*p* = 0.39). Faculty described research impact beyond traditional scholarly metrics, including clinical practice, research and practice directions, education, capacity building, knowledge mobilization and engagement.

**Discussion:**

This study provides a contemporary national characterization of research productivity and impact among rehabilitation science faculty in Canada. Findings support using bibliometric and grant funding indices as a component of research performance evaluations, emphasizing the importance of contextual interpretation and broadly conceptualizing research impact.

## Introduction

Research performance evaluations are important within academia to inform decisions related to hiring, promotion and tenure. These evaluations commonly use quantitative measures of research productivity and impact, such as bibliometric indices (e.g., publication counts, h-index) and success in obtaining competitive grant funding [1–4]. Understanding typical ranges in these measures and how they vary across academic ranks can support interpretation of research performance. For example, prior research has demonstrated that bibliometric indices generally increase from Assistant to Associate to Full Professor and that h-index is associated with markers of academic recognition (e.g., awards), supporting their use of as a component of research performance evaluation [4–7]. However, these quantitative indices have recognized limitations that make direct comparisons challenging, such as differences between research fields in approaches to conducting research, as well as publication and citation practices [8,9].

Differences in research and publishing practices across research fields requires discipline-specific understanding of research productivity and impact [4,5]. In rehabilitation sciences, MacDermid et al used data collected in 2013 to demonstrate research productivity and impact of physical therapy and occupational therapy (PT and OT) faculty across Canada, with median h-indices ranging from 5 among Assistant Professors to 18 among Full Professors and median Canadian Institute of Health Research (CIHR) grant funding as principal investigator (PI) ranging from $11,733 to $199,259, respectively [4]. Similar h-index results were reported in a study that used data collected in 2015 to characterize communication sciences and disorders (CSD) faculty in Canada and the United States [5]. Both studies emphasized the importance of interpreting research productivity and impact indices relative to academic rank and discipline. These studies were limited to tenure-track appointments and therefore did not capture the research productivity and impact of teaching-focused academic appointments, such as Professors of Teaching, where less time allocated to research and role expectations (e.g., educational leadership) may influence research productivity and impact [10,11].

Research productivity and impact are also influenced by contextual factors at the institutional level. Specifically, factors within the research environment have been associated with research productivity and impact, including institutional research strategy that provides direction and coordination for research activity, trainees who expand research capacity, resources that provide research facilities and funding, and supports to enable research such as protected time [1,4,12]. Examining associations between these contextual factors and measures of research productivity and impact is therefore important to support accurate interpretation of quantitative research performance evaluation metrics [10].

While quantitative bibliometric and grant funding indices of research productivity and impact can be useful for research performance evaluations, they provide a limited view of research impact. This limitation is particularly relevant in applied health disciplines such as rehabilitation sciences, where research may influence clinical practice, education and policy in ways that are not fully captured by quantitative bibliometric and grant funding indices [13]. Contemporary models of research assessment therefore advocate for evaluating impact across multiple dimensions, such as knowledge generation, development of people and collaborations, research community advancements, and contributions to society [14]. However, it is unknown how rehabilitation science researchers qualitatively describe the impact of their research. Understanding these perspectives can be useful to inform research performance evaluations in rehabilitation sciences.

Existing studies reflect the rehabilitation sciences research landscape of the mid-2010s [4,5]. Over the past decade, this landscape has evolved, with increases in the number of annual publications, greater emphasis on team-based research, and interdisciplinary collaborations [15–17]. These changes suggest that bibliometric and grant funding profiles of rehabilitation science faculty across Canada have also likely evolved. However, there are no contemporary national data characterizing research productivity and impact across PT, OT and CSD faculty in Canada, which can support accurate interpretation and classification during hiring, promotion, and tenure decisions or any associated appeals, or when assigning awards of merit.

### Aim

To characterize research productivity and impact of rehabilitation science (PT, OT, CSD) faculty across Canada.

### Objectives

1. To evaluate indices of research productivity and impact
2. To identify factors associated with research productivity and impact
3. To describe faculty perspectives on their research impact

For objective 1, we hypothesized that bibliometric and grant funding indices would be higher among faculty at more senior academic ranks (e.g., Full Professor) and in research-focused appointments (Assistant, Associate, Full Professors). For objective 2, we hypothesized that h-index and grant funding would be positively associated with each other, publication count, academic appointment (senior ranks, research-focused) and academic unit research environment characteristics (number of research supports, research trainees, academic unit grant funding). For objective 3, no hypothesis was generated owing to the qualitative focus.

## Methods

### Design

This mixed methods cross-sectional observational study used publicly available data and descriptive online surveys to characterize research productivity and impact of rehabilitation science faculty across Canada. Reporting aligns with STROBE (Strengthening of Reporting of Observational Studies in Epidemiology), CROSS (Consensus-based Checklist for Reporting Survey Studies) and CHERRIES (Checklist for Reporting Results of Internet E-Surveys) guidelines (Supplement 1) [18–20].

### Ethics

This study was approved by the Western University Non-Medical Research Ethics Board (ID: 124384). Participation in the survey was voluntary, and participants indicated their consent to participate by submitting a survey response. No incentives were offered to participate. Recruitment emails contained an attached Letter of Information describing the study, described measures to ensure anonymity (e.g., username and password protected access limited to research team, de-identified data download, de-identified storage on secure password protected server), a reminder of the right to withdraw at any time, a copy of survey questions and a link to complete the survey.

### Setting

Publicly available sources were used to extract academic appointment, bibliometric and grant funding data for all full-time PT, OT and CSD faculty in Canada. Two online surveys were developed using REDCap (Research Electronic Data Capture) electronic data capture tools hosted by the National Centre for Audiology at Western University. REDCap is a secure web-based software platform designed to support data capture for research studies [21,22]. Surveys collected data from Heads of PT, OT and CSD academic units about the research environment in the unit they lead and from faculty about their self-described research impact. Online surveys facilitated broad data collection independent of geography and flexibility for participants when responding [23].

### Participants and recruitment

#### Participants with publicly available data.

All full-time faculty listed on PT, OT, CSD Canadian academic unit websites were eligible to have their publicly available data extracted from websites. Canadian academic units were identified from each discipline’s accrediting agency website (PT: www.peac-aepc.ca; OT: www.caot.ca; CSD: https://www.cacup-aslp.ca).

### Survey participants

All Heads of PT, OT and CSD academic units across Canada were eligible to participate in the Heads survey. All full-time faculty members employed in PT, OT and CSD academic units across Canada were eligible to participate in the faculty survey. Heads and faculty were excluded if they were not able or willing to provide consent and not fluent in written English or French language.

### Survey recruitment

Using a total population sampling approach, [24] Heads of academic units and full-time faculty were invited to participate through recruitment emails using a two-staged approach. In the first stage, Heads of academic units were invited to participate in the Heads survey. In the second stage, Heads of academic units were invited to participate in the full-time faculty survey with a request to forward the invitation to the full-time faculty in the academic unit they lead. In both stages, two reminder emails were sent at two-week intervals to promote participation [25].

### Data sources and variables

#### Full-time faculty data from academic unit websites.

Data were extracted from academic unit websites for full-time faculty members March – April 2024, including:

- Name
- Email address
- Discipline
- Appointment title and stage of career (e.g., Instructor, Assistant Professor)
- Administrative or research chair roles, as applicable (e.g., Head of unit)

Heads of academic units and full-time faculty were classified into PT, OT or CSD disciplines based on the unit in which they are listed on university websites, professional designation, degrees awarded and survey response. Faculty were classified as Rehabilitation Scientists if it was not possible to determine their discipline.

### Bibliometric data

All full-time faculty identified from academic unit websites were searched in the freely available Publish or Perish software April – June 2024 [26]. Publish or Perish searches and retrieves citations from search engines (e.g., Google Scholar) to calculate bibliometric indices of research productivity and impact. Google Scholar retrieves citations from the broadest range of document types (e.g., conference proceedings, book chapters), [27] which are important mechanisms for rehabilitation science researchers to impact clinical practice and policy. While Google Scholar is not without limitations (e.g., proprietary search strategy and coverage), [27,28] Google Scholar Profile can mitigate this limitation as it allows researchers to curate a list of their publications, facilitating more accurate search retrieval and citation attribution. Therefore, to enable the most comprehensive bibliometric analysis, [29] faculty names were searched in Publish or Perish first using Google Scholar Profile. If a Google Scholar Profile did not exist, faculty names were then searched using Google Scholar.

Publish or Perish calculates a range of bibliometric indices. Number of publications is an indication of research productivity while citation counts provide an index of research impact, as a high citation count can suggest a scientist’s research is being used to advance scholarly knowledge [3,30]. The h-index, derived from number of publications and citations, indicates that an author has published *h* articles that have each received *h* citations (e.g., h-index of 10 indicates an author has published at least 10 articles that have each received at least 10 citations) [31]. It provides a broad estimate of cumulative research impact and is the most common bibliometric index of research productivity and impact [2,3,31,32]. The g-index also considers publication and citation count, however in contrast to the h-index it gives greater weight to highly cited articles, calculated as the number of most-cited publications whose combined citation count is at least equal to the square of that number (e.g., g-index of 10 indicates an author’s 10 most-cited publications have received at least 100 citations in total) [30]. The following data were extracted from Publish or Perish:

- Publication count
- Citation count
- h-index
- g-index

To ensure accurate author attribution, citations contributing to the h-index and the subsequent 20 highly cited publications were examined in detail. Targeted author full name, co-authors, academic affiliation, title, abstract and research content of the full manuscript were evaluated to determine accurate attribution; incorrect author attributions were removed. See Supplement 2 for more details on the data extraction protocol.

### Tri-Council grant funding data

All PT, OT and CSD full-time faculty identified from academic unit websites were searched in publicly available research grant funding databases from CIHR (https://webapps.cihr-irsc.gc.ca/decisions), Natural Sciences and Engineering Research Council (NSERC; https://www.nserc-crsng.gc.ca/ase-oro/index_eng.asp) and Social Sciences and Humanities Research Council (SSHRC; http://www.outil.ost.uqam.ca/crsh/rechproj.aspx). Owing to differences in reporting across Tri-Council databases, steps were taken to standardize data extracted. Each Tri-Council database has a different year of inception (CIHR: 2009, NSERC: 1991, SSHRC: 1998), so all databases were searched from 2009 to July 2024 fiscal year. Funding data was extracted for successful grants as PI (CIHR), Project lead (NSERC) and Applicant (SSHRC). Funding awarded as Co-PI in CIHR was not extracted because the equivalent could not be extracted in NSERC and SSHRC. Databases also report grant successes differently (CIHR: Awarded, NSERC: Funded, SSHRC: Funded) so steps were taken to standardize grant amount awarded because this is typical reporting for faculty (e.g., in curriculum vitae). After identifying SSHRC grants funded, SSHRC provided grant amount awarded. NSERC indicated grant awarded information was confidential and therefore could not provide this information. Instead, NSERC grant installments were summed to calculate the total amount awarded per grant. For recently awarded NSERC grants, consistent future installment funding was assumed and added to estimate total amount awarded. Additional decision making related to data extracted can be found in Supplement 2. From each Tri-Council database, data obtained included:

- Number of grants
- Amount awarded per grant

### Heads of academic units survey

The United Kingdom’s 2021 Research Excellence Framework [33] was used to inform development of the Heads of academic units survey, to collect research environment data. Questions were optimized through consultation with the study team, which included methodological expertise in survey development and leadership of rehabilitation science academic units internationally. The Heads survey was piloted with two Heads of academic units within the research team to ensure conciseness, clarity and quality of survey questions. Following the pilot, question structure was simplified to enhance feasibility and completeness of data collected. Pilot survey responses were not used for data analysis and pilot participants were not excluded from completing the definitive survey. The Heads survey collected data May – September 2024 and included 5 sections, with each section on a different survey page:

1. Profile of faculty in unit
2. Research support
3. Research facilities
4. Research funding
5. Trainees

Survey questions comprised of both quantitative open and closed questions, as well as qualitative questions that provided participants with the opportunity to elaborate on prior responses. A back button enabled respondents to return to prior survey pages if needed. At the end of the survey, Heads were asked to provide the name of the University where they work and select the discipline of the unit they lead, to enable linking of academic unit research environment data with publicly available data. When duplicate academic unit responses occurred, participants were contacted to confirm which response should be used for analysis. The complete Heads survey can be found in Supplement 3.

### Full-time faculty survey

Rehabilitation science faculty self-described research impact was obtained from a larger cross-sectional survey that included sections on University appointment details, research grant funding, supervision, research impact and demographics. The current study used only the research impact section. Findings from the wider full-time faculty survey will be published separately. The research impact section consisted of a single open-ended qualitative question (“Please describe or list the impact of your program of rehabilitation science research”). The question was prefaced to provide broad definitions of research using Boyer’s framework and impact using an adapted description of the UK’s Research Excellence Framework [33,34]. If a faculty member indicated in the survey they have published research or have been awarded Tri-Council grant funding using a different name than that listed on their university website, bibliometric and grant funding data associated with the previous name(s) were searched, extracted and amalgamated with that extracted using the name listed on the university website. The faculty survey collected data from July – October 2024.

### Bias

To reduce information bias, [18,35] data extracted from publicly available websites was completed in accordance with an established protocol (Supplement 2) by research assistants who do not have university faculty appointments (LT, SA, DP, LM). For French-speaking Universities, a member of the research team fluent in French extracted data (LT, SA). After a period of training, data extraction was piloted independently in duplicate on five faculty members. Adjustments were made to ensure standardized data extraction procedures. Data extraction was conducted by three researchers (SA, LM, DP) and supported by a second researcher (LT), who independently checked accuracy of extraction of a subset of data. Owing to the challenging nature of the data sources (e.g., heterogeneity of grant funding reporting in Tri-Council databases, faculty without curated Google Scholar Profiles), inconsistencies were identified in the subset leading to a third research team member re-analyzing / extracting data (LT, KK). Differences in data extraction were then resolved through discussion of issues with the research team.

### Sample size

Website, bibliometric and grant funding data were extracted for all full-time faculty identified on academic unit websites. However, it was anticipated that not all Heads of academic units and full-time faculty would respond to surveys. Dillman’s equation was used to estimate precision in survey samples, using 95% confidence interval and 50% probability to respond or not [36,37]. Assuming n = 41 Heads of academic units in Canada based on number of professional programs on accrediting agency websites, 37 Heads survey responses would ensure representation of the population. From the 685 full-time faculty identified on websites, 246 faculty survey responses would ensure representation.

### Data analysis

For objective 1, descriptive statistics were used to characterize research environment data as well as bibliometric and grant funding indices of research productivity and impact. Owing to positively skewed data, heterogeneity of variance and unequal samples across groups, the Kruskal-Wallis test compared differences in bibliometrics and grant funding indices by appointment title [38]. Dunn’s post-hoc test evaluated pairwise comparisons between appointment titles, using Bonferroni correction to control Type I errors [39].

For objective 2, Spearman correlations examined univariate associations among bibliometric indices and grant funding, which were interpreted using conventional coefficient thresholds (negligible = 0.00–0.10, weak = 0.10–0.39, moderate = 0.40–0.69, strong = 0.70–0.89, very strong = 0.90–1.0) [40]. General linear models explored associations with h-index and sum of Tri-Council grant funding awarded as PI (CIHR), Project Lead (NSERC) and Applicant (SSHRC) for the total population of faculty (n = 685). Variable selection was informed by MacDermid et al.[4] and univariate correlations to enable comparisons with prior research and minimize multicollinearity. Models for h-index included discipline, appointment title, publication count, and sum of Tri-Council grant funding awarded. Models for Tri-Council grant funding awarded included discipline, appointment title, h-index and publication count. To explore clustering within academic units, linear mixed effects models were then fitted for h-index and sum of Tri-Council grant funding awarded using restricted maximum likelihood estimation [41]. Academic unit was specified as a random effect intercept, while fixed effects remained the same as those included in the general linear models. Clustering by academic unit was examined using intraclass correlation coefficients (ICC) and the Wald test to assess between-unit variability [42]. When clustering by academic unit was observed, unit-level research environment characteristics (research supports, number of trainees, unit research funding over the past five years) were examined in the subset of faculty with complete data from the Heads of academic units survey (n = 401 faculty across 22 units) to explore whether between-unit variability could be explained by the research environment. This was evaluated using a linear multilevel mixed effects model, retaining academic unit as a random intercept [42]. Owing to positively skewed data, continuous variables were natural logarithm +1 transformed to accommodate zero values and improve linearity and heteroscedasticity [43]. Log–log models were used and heteroskedasticity-consistent (HC3) robust standard errors are reported [43,44]. All analyses were conducted in Microsoft Excel and SPSS (version 30; IBM Corp., Armonk, NY). Statistical significance was set at *p* < 0.05.

For objective 3, self-described research impact was analyzed using an inductive approach to content analysis [45,46]. Survey responses written in French were translated by a French speaking member of the research team (SA). After translation, all responses were read multiple times and open coded, line by line in English language using Microsoft Word and Excel by one researcher (KK). Codes were structured into hierarchical descriptive themes and subthemes of self-described research impact [46].

## Results

### Rehabilitation science faculty characteristics

A total of 41 rehabilitation science academic units (PT = 15, OT = 14, CSD = 12) were identified on accrediting agency websites. Across these units, 685 full-time rehabilitation science faculty members were identified from university websites, comprising PT (n = 262; 38%), OT (n = 248; 36%), CSD (n = 168; 25%) and Rehabilitation Scientists (n = 7; 1%). The largest proportion held Full Professor (28%), Associate Professor (26%) and Assistant Professor (21%) appointments. Smaller numbers of faculty held Professor of Teaching across all ranks (12%) and Instructor appointments (13%). The distribution by discipline is shown in Table 1.

**Table 1:** Distribution of full-time rehabilitation science faculty by appointment.

Appointment (n)	All disciplines	PT	OT	CSD	Rehabilitation scientist
Assistant Professor	143	50	55	36	2
Associate Professor	181	64	71	44	2
Full Professor	191	69	63	57	2
Assistant Professor, Teaching	43	20	18	5	–
Associate Professor, Teaching	26	11	8	7	–
Full Professor, Teaching	13	6	5	2	–
Instructor	88	42	28	17	1

### Research environment characteristics

A total of 36 unique survey responses from Heads of academic unit were received, with 1 response excluded from analysis as only the first 2 survey questions were answered. This resulted in 35 responses contributing research environment data across 15 of 15 PT (100%), 11 of 14 OT (79%), 7 of 12 CSD (58%) and 2 unknown discipline academic units in Canada. CSD responses included units offering only Speech and Language Pathology (SLP; n = 5) and both SLP and Audiology (n = 2). Complete data were obtained from 63% of survey responses from Heads of academic units. Sample sizes reported for individual variables reflect the number of survey responses received, with remaining data missing because items were skipped in the survey. Research environment descriptive statistics are reported in the main text for the whole sample and select discipline-specific data; additional discipline-specific data are provided in Supplement 4.

### Research support

Across 35 academic units, most had administrative grant support at the unit level pre-award (mean 97% overall [100% PT, 100% OT, 85% CSD]) and post-award (69% [80% PT, 64% OT, 57% CSD]), research labs within the academic unit (83% [87% PT, 82% OT, 86% CSD]) or within a hospital (71% [80% PT, 64% OT, 57% CSD]), and a research strategic plan (60% [73% PT, 46% OT, 57% CSD]). Fewer reported having knowledge translation support (37% [53% PT, 27% OT, 14% CSD]) and research labs within community organizations (34% [33% PT, 55% OT, 0% CSD]). Additional support (29% [40% PT, 27% OT, 0% CSD]) included university research institutes and centers, research labs within the university, research mentorship, and teaching buyout to increase research time.

### Research facilities

Across 32 academic units, Heads reported nearly all faculty (mean 97% overall [100% PT, 90% OT, 100% CSD]) had the research facilities needed to conduct research. Access to specific research facilities was reported by 34 academic units. Variability was seen across responses as indicated by wide IQRs and a U-shaped, bimodal distribution with a high percentage of access near 0 and 100%. The median (IQR [discipline median]) percentage of faculty within an academic unit who had their own research lab space was 39% (11–99% [30% PT, 30% OT, 100% CSD]), access to shared research lab space was 26% (4–98% [27% PT, 50% OT, 2% CSD]), research conducted in a hospital was 22% (8–40% [27% PT, 10% OT, 10% CSD]), and in collaboration with community organizations was 31% (11–82% [45% PT, 25% OT, 20% CSD]). Facilities were not mutually exclusive so heads could report faculty access to more than one facility.

### Research type

Across 33 academic units, Heads reported that faculty engaged in a range of research types. The largest median (IQR [discipline median]) proportion was clinical research at 40% (30–60% [49% PT, 29% OT, 60% CSD]), followed by health services research at 10% (4–20% [14% PT, 9% OT, 10% CSD]) and basic science at 10% (0–15% [10% PT, 0% OT, 15% CSD]). Other research types (e.g., social sciences) accounted for smaller proportions (<5%).

### Research funding in past 5 years

Across 24 academic units, the largest median (IQR [discipline median]) share of research funding in the past 5 years came from CIHR or international equivalents at 31% (11–48% [42% PT, 24% OT, 5% CSD]), followed by National targeted funding at 16% (7–28% [20% PT, 32% OT, 4% CSD]) and Provincial agencies or international equivalents at 8% (4–13% [9% PT, 5% OT, 12% CSD]). Other funding sources (e.g., SSHRC, Internal funds) each accounted for ≤6%. In the 22 academic units that reported total grant funding received in the past 5 years, median (IQR [discipline median]) funding was $6,843,668 ($3,241,717–$19,252,619 [$15,188,246 PT, $4,456,453 OT, $3,492,178 CSD]) and ranged from $110,000 – $135,684,716.

### Trainees

Across 30 academic units, Heads reported a total of 1,284 trainees: 620 PhD students, 517 MSc students, and 147 postdoctoral trainees. Per academic unit, the median (IQR [discipline median]) number of current trainees was: PhD students 18 (12–26 [15 PT, 18 OT, 16 CSD]), MSc students 13 (8–17 [15 PT, 13 OT, 9 CSD]), and postdoctoral trainees 4 (2–6 [5 PT, 5 OT, 2 CSD]), corresponding to 37 (25–50 [38 PT, 42 OT, 23 CSD]) total trainees per academic unit.

### Bibliometric and grant funding indices of research productivity and impact

Descriptive statistics of bibliometric and grant funding indices of research productivity and impact for all full-time rehabilitation science faculty across all appointments are presented in Table 2. PT, OT and CSD-specific bibliometric and grant funding descriptive statistics are presented in Tables 3, 4 and 5, respectively. Across all rehabilitation science faculty and within each discipline, median values for most indices increased with appointment rank and were highest for Full Professors, while values were consistently lower for Professors of Teaching and Instructors. Supplement 5 contains the full results of Kruskall-Wallis tests comparing differences between appointment titles for all rehabilitation science faculty combined.

**Table 2:** Bibliometric and grant funding indices of research productivity and impact by appointment title for all full-time rehabilitation science faculty (PT, OT, CSD combined) across all appointments.

Index	Instructor	Assistant Professor, Teaching	Associate Professor, Teaching	Full Professor, Teaching	Assistant Professor	Associate Professor	Full Professor
**Publication count** Median (IQR) Mean (SD)	2 (0-6) 8 (23)	4 (1–13) 17 (46)	6 (3–19) 13 (16)	7 (3–30) 15 (15)	30 (16–60) 45 (42)	71 (46–105) 89 (71)	131 (79–202) 159 (129)
**Citation count** Median (IQR) Mean (SD)	9 (0–97) 183 (710)	53 (0–164) 137 (247)	114 (22–345) 296 (525)	226 (3–674) 482 (722)	504 (195–1,050) 907 (1,524)	1624 (872–2,841) 2,741 (5,239)	3,990 (1,618–7,119) 6,178 (8,948)
**h-index** Median (IQR) Mean (SD)	1 (0–3) 3 (5)	2 (0–5) 4 (4)	3 (2–7) 5 (5)	4 (1–12) 6 (6)	9 (6–16) 12 (8)	19 (14–25) 20 (9)	32 (21–42) 33 (18)
**g-index** Median (IQR) Mean (SD)	2 (0–5) 4 (10)	4 (0–9) 6 (8)	6 (3–15) 9 (10)	6 (1–22) 11 (12)	19 (13–31) 23 (16)	38 (27–52) 42 (24)	59 (38–82) 65 (39)
**Tri-Council: No. grants awarded (n)** Median (IQR) Mean (SD)	0 (0–0) 0 (0)	0 (0–0) 0 (0)	0 (0–0) 0 (0)	0 (0–0) 0 (0)	0 (0–1) 1 (1)	1 (0–3) 2 (3)	2 (0–5) 4 (5)
**Tri-Council: Funds awarded ($)** Median (IQR) Mean (SD)	0 (0–0) 1,855 (15,020)	0 (0–0) 19,264 (126,324)	0 (0–0) 23,629 (120,484)	0 (0–0) 0 (0)	0 (0–130,050) 458,652 (2,082,375)	100,000 (0–1,012,129) 1,282,553 (44,53,874)	365,096 (0–1,533,001) 2851481 (9854862)
**CIHR: No. grants awarded as PI (n)** Median (IQR) Mean (SD)	0 (0−0) 0 (0)	0 (0−0) 0 (0)	0 (0−0) 0 (0)	0 (0−0) 0 (0)	0 (0-1) 0 (1)	0 (0-2) 2 (3)	1 (0-3) 3 (5)
**CIHR: Funds awarded as PI ($)** Median (IQR) Mean (SD)	0 (0−0) 0 (0)	0 (0−0) 19,264 (126,324)	0 (0−0) 23,629 (120,484)	0 (0−0) 0 (0)	0 (0-74,413) 429,672 (2,083,227)	0 (0–908,613) 1,226,063 (4,456,605)	66,461 (0–1,400,969) 2,477,473 (8,487,454)

All data are positively skewed; median (IQR) are most appropriate numbers to interpret. All full-time faculty with administrative or chair roles are considered within their academic appointment. Tri-Council represents the sum of grants awarded as PI (CIHR), Project Lead (NSERC), and Applicant (SSHRC) from 2009 to July 2024 fiscal year.

**Table 3:** Bibliometric and grant funding indices of research productivity and impact by appointment title for all PT faculty across all appointments.

Index	Instructor	Assistant Professor, Teaching	Associate Professor, Teaching	Full Professor, Teaching	Assistant Professor	Associate Professor	Full Professor
**Publication count** Median (IQR) Mean (SD)	2 (0–5) 11 (31)	6 (2–13) 9 (10)	17 (5–35) 22 (20)	16 (1–33) 17 (17)	44 (17–66) 52 (50)	92 (52–140) 113 (91)	175 (99–242) 212 (161)
**Citation count** Median (IQR) Mean (SD)	5 (0–100) 288 (1011)	55 (0–105) 92 (134)	196 (56–460) 502 (730)	491 (0–912) 546 (606)	522 (179–1,261) 1,213 (2,259)	1,865 (972–4,407) 3,996 (6,594)	5,594 (3,446–9,706) 9,299 (12,435)
**h-index** Median (IQR) Mean (SD)	1 (0–2) 3 (7)	3 (0–5) 3 (3)	4 (3–12) 8 (7)	7 (0–14) 7 (7)	10 (6–17) 13 (10)	23 (15–32) 24 (12)	40 (25–50) 41 (19)
**g-index** Median (IQR) Mean (SD)	2 (0–4) 6 (13)	2 (0–4) 6 (6)	7 (5–21) 14 (12)	12 (0–25) 12 (12)	21 (11–35) 26 (21)	43 (31–66) 50 (29)	71 (55–97) 82 (44)
**Tri-Council: No. grants awarded (n)** Median (IQR) Mean (SD)	0 (0–0) 0 (0)	0 (0–0) 0 (1)	0 (0–0) 0 (1)	0 (0–0) 0 (0)	0 (0–1) 1 (1)	2 (0–4) 3 (4)	3 (1–7) 5 (7)
**Tri-Council: Funds awarded ($)** Median (IQR) Mean (SD)	0 (0–0) 0 (0)	0 (0–0) 41,418 (185,228)	0 (0–0) 55,850 (185,233)	0 (0–0) 0 (0)	0 (0–134,832) 876,837 (3,378,970)	191,000 (0–1,460,732) 1,934,637 (6,463,703)	844,998 (22,237–2,569,613) 4,190,736 (9,983,353)
**CIHR: No. grants awarded as PI (n)** Median (IQR) Mean (SD)	0 (0–0) 0 (0)	0 (0–0) 0 (1)	0 (0–0) 0 (1)	0 (0–0) 0 (0)	0 (0–1) 1 (1)	2 (0–3) 3 (4)	3 (0–6) 4 (7)
**CIHR: Funds awarded as PI ($)** Median (IQR) Mean (SD)	0 (0–0) 0 (0)	0 (0–0) 41,418 (185,228)	0 (0–0) 55,850 (185,233)	0 (0–0) 0 (0)	0 (0–130,050) 852,224 (3,380,496)	112,485 (0–1,215,236) 1,887,317 (6,462,541)	736,252 (0–2,548,768) 4,090,771 (9,958,499)

All data are positively skewed; median (IQR) are most appropriate numbers to interpret. All full-time faculty with administrative or chair roles are considered within their academic appointment. Tri-Council represents the sum of grants awarded as PI (CIHR), Project Lead (NSERC), and Applicant (SSHRC) from 2009 to July 2024 fiscal year.

**Table 4:** Bibliometric and grant funding indices of research productivity and impact by appointment title for all OT faculty across all appointments.

Index	Instructor	Assistant Professor, Teaching	Associate Professor, Teaching	Full Professor, Teaching	Assistant Professor	Associate Professor	Full Professor
**Publication count** Median (IQR) Mean (SD)	3 (0–7) 7 (16)	5 (3–32) 31 (70)	8 (2–11) 8 (7)	5 (3–19) 9 (11)	30 (18–58) 43 (35)	69 (49–98) 86 (63)	126 (65–186) 143 (107)
**Citation count** Median (IQR) Mean (SD)	34 (0–105) 90 (139)	69 (9–301) 223 (339)	120 (27–291) 200 (265)	48 (16–280) 128 (145)	491 (202–1,050) 749 (947)	1,272 (861–2,515) 2,429 (5,314)	3,046 (1,018–6,287) 4,806 (6,256)
**h-index** Median (IQR) Mean (SD)	2 (0–3) 3 (3)	4 (1–8) 5 (5)	4 (2–6) 4 (4)	4 (2–7) 4 (4)	9 (7–15) 11 (6)	18 (14–23) 19 (7)	26 (14–38) 28 (17)
**g-index** Median (IQR) Mean (SD)	3 (0–6) 4 (5)	5 (2–15) 9 (9)	7 (2–11) 8 (7)	5 (2–12) 7 (7)	19 (13–31) 22 (14)	34 (28–47) 40 (21)	46 (28–78) 56 (37)
**Tri-Council: No. grants awarded (n)** Median (IQR) Mean (SD)	0 (0–0) 0 (0)	0 (0–0) 0 (0)	0 (0–0) 0 (0)	0 (0–0) 0 (0)	0 (0–2) 1 (2)	1 (0–4) 2 (3)	2 (0–4) 3 (4)
**Tri-Council: Funds awarded ($)** Median (IQR) Mean (SD)	0 (0–0) 861 (4,555)	0 (0–0) 0 (0)	0 (0–0) 0 (0)	0 (0–0) 0 (0)	0 (0–218,025) 344,047 (863,965)	258,215 (0–1,248,841) 1,374,951 (3,477,638)	224,954 (0–1,218,434) 3,522,869 (13,420,789)
**CIHR: No. grants awarded as PI (n)** Median (IQR) Mean (SD)	0 (0–0) 0 (0)	0 (0–0) 0 (0)	0 (0–0) 0 (0)	0 (0–0) 0 (0)	0 (0–1) 1 (1)	1 (0–3) 2 (3)	1 (0–3) 2 (4)
**CIHR: Funds awarded as PI ($)** Median (IQR) Mean (SD)	0 (0–0) 0 (0)	0 (0–0) 0 (0)	0 (0–0) 0 (0)	0 (0–0) 0 (0)	0 (0–115,000) 309,256 (863,433)	25,000 (0–1,248,841) 1,319,753 (3,486,821)	124,882 (0–1,009,636) 2,760,769 (10,190,964)

All data are positively skewed; median (IQR) are most appropriate numbers to interpret. All full-time faculty with administrative or chair roles are considered within their academic appointment. Tri-Council represents the sum of grants awarded as PI (CIHR), Project Lead (NSERC), and Applicant (SSHRC) from 2009 to July 2024 fiscal year.

**Table 5:** Bibliometric and grant funding indices of research productivity and impact by appointment title for all CSD faculty across all appointments.

Index	Instructor	Assistant Professor, Teaching	Associate Professor, Teaching	Full Professor, Teaching	Assistant Professor	Associate Professor	Full Professor
**Publication count** Median (IQR) Mean (SD)	1 (0–4) 2 (3)	0 (0–1) 0 (0)	3 (1–5) 4 (5)	21 (4–*) 21 (24)	22 (15–44) 35 (32)	51 (40–75) 60 (29)	99 (66–145) 114 (78)
**Citation count** Median (IQR) Mean (SD)	1 (0–71) 42 (73)	0 (0–10) 4 (8)	15 (2–29) 83 (184)	1,174 (5–*) 1,174 (1,653)	366 (244–875) 687 (737)	1,491 (598–1,914) 1,443 (988)	2,903 (1,440–5,099) 3,964 (4,607)
**h-index** Median (IQR) Mean (SD)	1 (0–2) 1 (1)	0 (0–1) 0 (0)	2 (1–3) 3 (3)	8 (1–*) 8 (10)	9 (5–15) 10 (6)	17 (11–22) 16 (6)	27 (19–37) 28 (13)
**g-index** Median (IQR) Mean (SD)	1 (0–4) 2 (3)	0 (0–1) 0 (0)	3 (1–3) 4 (5)	20 (1–*) 20 (26)	18 (12–27) 21 (13)	34 (23–42) 33 (14)	51 (36–66) 53 (29)
**Tri-Council: No. grants awarded (n)** Median (IQR) Mean (SD)	0 (0–0) 0 (0)	0 (0–0) 0 (0)	0 (0–0) 0 (0)	0 (0–0) 0 (0)	0 (0–1) 1 (1)	1 (0–1) 1 (1)	2 (0–4) 3 (3)
**Tri-Council: Funds awarded ($)** Median (IQR) Mean (SD)	0 (0–0) 8,183 (33,737)	0 (0–0) 0 (0)	0 (0–0) 0 (0)	0 (0–0) 0 (0)	0 (0–58,056) 61,624 (132,888)	7,499 (0–146,250) 177,402 (395,320)	265,000 (0–725,207) 499,662 (838,371)
**CIHR: No. grants awarded as PI (n)** Median (IQR) Mean (SD)	0 (0–0) 0 (0)	0 (0–0) 0 (0)	0 (0–0) 0 (0)	0 (0–0) 0 (0)	0 (0–0) 0 (0)	0 (0–0) 0 (1)	0 (0–1) 1 (2)
**CIHR: Funds awarded as PI ($)** Median (IQR) Mean (SD)	0 (0–0) 0 (0)	0 (0–0) 0 (0)	0 (0–0) 0 (0)	0 (0–0) 0 (0)	0 (0–0) 33,846 (114,461)	0 (0–0) 108,077 (378,012)	0 (0–35,871) 212,228 (683,289)

* = sample size too small to calculate (n = 2 Full Professors of Teaching in CSD). All data are positively skewed; median (IQR) are most appropriate numbers to interpret. All full-time faculty with administrative or chair roles are considered within their academic appointment. Tri-Council represents the sum of grants awarded as PI (CIHR), Project Lead (NSERC), and Applicant (SSHRC) from 2009 to July 2024 fiscal year.

For all rehabilitation science faculty combined, Full Professors had significantly higher median publication count (n = 131) and h-index (n = 32) than all other appointment titles (*p* < 0.001 for all pairwise comparisons, Table 2, Supplement 5), including Associate Professors (publication count: 71, h-index: 19), Assistant Professors (publication count: 30, h-index: 9), Full Professors of Teaching (publication count: 7, h-index: 4), Associate Professors of Teaching (publication count: 6, h-index: 3), Assistant Professors of Teaching (publication count: 4, h-index: 2) and Instructors (publication count: 2, h-index: 1). Associate Professors had significantly higher publication count and h-index than Assistant Professors, Professors of Teaching at all ranks, and Instructors (*p* < 0.001). Assistant Professors had significantly higher publication count than Instructors and Professors of Teaching at Assistant and Associate ranks (*p* ≤ 0.01). Although median publication count was higher for Assistant Professors than Full Professors of Teaching, this difference was not significant (*p* = 0.40). Assistant Professors also had significantly higher h-index than Instructors and Assistant Professors of Teaching (*p* < 0.001). Although median h-index was higher for Assistant Professors than Professors of Teaching at Associate and Full ranks, these differences were not significant (*p* ≥ 0.08). Publication count and h-index did not significantly differ between Professors of Teaching at all ranks and Instructors (*p* = 1.0). This pattern of publication count and h-index was similar within each discipline (Supplement 5).

For all rehabilitation science faculty combined, Full Professors were awarded a significantly greater median number of Tri-Council grants (n = 2) and funding (n = $365,096) than all other appointment titles (*p* < 0.001), except Associate Professors where differences were not significant despite higher median values (1 grant, $100,000 funding, *p* ≥ 0.06; Table 2, Supplement 5). Assistant Professors, Professors of Teaching across all ranks and Instructors had median values of 0 for both Tri-Council grant count and funding. Associate Professors were awarded a significantly greater number of Tri-Council grants and grant funds than Assistant Professors, Professors of Teaching at all ranks, and Instructors (*p* ≤ 0.001). Assistant Professors were awarded a significantly greater number of Tri-Council grants and grant funds than Instructors and Assistant Professors of Teaching (*p* ≤ 0.006), and similar grant number and funds as Full and Associate Professors of Teaching (*p* ≥ 0.07). Number of Tri-Council grants and grant funds did not significantly differ between Professors of Teaching at all ranks and Instructors (*p* = 1.0). This pattern of Tri-Council grants and funding was similar within each discipline (Supplement 5).

### Associations with research productivity and impact

Univariate correlations showed that all bibliometric and grant funding indices were significantly and positively correlated (Table 6). Bibliometric indices were very strongly correlated with each other, while correlations between publication and funding were moderate.

**Table 6:** Univariate correlations between bibliometric and grant funding indices for all full-time rehabilitation science faculty (PT, OT, CSD combined) across all appointments.

Index	h-index	g-index	Publication count	Citation count	CIHR funding
g-index	0.98*	–	–	–	–
Publication count	0.94*	0.93*	–	–	–
Citation count	0.97*	0.98*	0.91*	–	–
CIHR funding	0.59*	0.58*	0.60*	0.57*	–
Tri-Council grant funding	0.65*	0.64*	0.67*	0.63*	0.86*

* *p* < 0.001. CIHR funding is amount awarded as PI. Tri-Council grant funding is the sum of the amount awarded from CIHR (as PI), NSERC (as Project Lead), and SSHRC (as Applicant).

### h-index

For the total population of faculty, h-index was associated with publication count (β = 0.62, 95%CI 0.60 to 0.65, *p* < 0.001), indicating that a 1% increase in publications was associated with an estimated 0.62% increased h-index, holding all other variables constant (Supplement 6). Appointment title was also associated with h-index (*p* < 0.001), with earlier career ranks and teaching focused appointments being associated with lower h-index values relative to Full Professors (e.g., Assistant Professors 14% (95%CI −20 to −8%) lower, Assistant Professors of Teaching 29% (95%CI −37 to −19%) lower). Discipline (*p* = 0.66) and sum of Tri-Council grant funding awarded (*p* = 0.32) were not associated with h-index.

Accounting for clustering of faculty within academic units did not meaningfully change associations. Publication count (β = 0.61, 95%CI 0.58 to 0.64, *p* < 0.001) and appointment title (*p <* 0.001) remained associated with h-index, while discipline (*p* = 0.77) and grant funding (*p* = 0.39) were not. The random effect of academic unit indicated that approximately 18.6% of the variability in h-index was attributable to differences between academic units (ICC = 0.186, *p* = 0.003; Supplement 6). For the subset of faculty with complete research environment data (n = 401 faculty across 22 academic units) that could be added to the model, 11% of variability in h-index was attributable to differences between academic units (ICC = 0.110, *p =* 0.07) with no significant associations between h-index and unit-level research environment characteristics, including research supports (*p* = 1.00), number of trainees (*p* = 0.71), and unit research funding over the past five years (*p* = 0.70; Supplement 6).

### Sum of Tri-Council grant funding awarded

For the total population of rehabilitation science faculty, sum of Tri-Council grant funding awarded was associated with publication count (β = 1.77, 95%CI 0.85 to 2.69, *p* < 0.001), indicating that a 1% increase in publications was associated with an estimated 1.77% increased Tri-Council grant funding awarded, holding all other variables constant (Supplement 6). Appointment title was also associated with Tri-Council grant funding awarded (*p* = 0.01), with most earlier career ranks and teaching focused appointments being associated with lower Tri-Council grant funding relative to Full Professors (e.g., Assistant Professors 83% (95%CI −95 to −38%) lower, Assistant Professors of Teaching 95% (95%CI −99 to −58%) lower). Discipline (*p* = 0.47) and h-index (*p* = 0.32) were not associated with Tri-Council grant funding. Accounting for clustering of faculty within academic units indicated minimal between-unit variability in funding (ICC = 0.016, *p* = 0.39, Supplement 6). Given this limited clustering, research environment characteristics were not further explored.

### Rehabilitation faculty’s self-described research impact

Of the 685 faculty identified on academic unit websites, 138 faculty (20%) responded to the faculty survey, of whom 72 provided qualitative descriptions of their self-described research impact. Survey responses reflected 35 PT, 18 OT, and 19 CSD faculty (n = 10 SLP, n = 9 Audiology). 6 research impact themes were identified, each with 3–4 subthemes, and all disciplines contributed to every theme:

1. Scholarly impact and recognition of excellence
2. Innovate and influence rehabilitation clinical practice
3. Shape rehabilitation research and practice directions
4. Elevate excellence in rehabilitation education
5. Build research capacity in rehabilitation research
6. Knowledge mobilization and engagement in rehabilitation science research

Table 7 includes rehabilitation faculty’s self-described research impact themes, their associated subthemes and illustrative quotes.

**Table 7:** Rehabilitation science faculty’s self-described impact of their program of research.

Research impact theme	Sub-themes	Illustrative quotes
Scholarly impact and recognition of excellence	Bibliometric indices	*“# Peer reviewed publications: 229 H-Index 75; citations. 20,593”* Full Professor, OT (participant 80)
	Research recognized for excellence through awards	*“I am listed in the Stanford-Elsevier’s top 2% of scientists in the world”* Full Professor, Audiology (participant 95)
	Research grant funding	*“Co-led CIHR-funded cohort focused on 3-10 year consequences and an Arthritis Society-funded inception cohort”* Associate Professor, PT (participant 40)
Innovate and influence rehabilitation clinical practice	Contribute to clinical practice guidelines and standards	*“I develop and implement clinical protocols and guidelines to support evidence-based infant hearing health systems. The protocols I have developed are used worldwide in early hearing loss detection and intervention programs”* Assistant Professor, Audiology (participant 26)
	Develop, implement and evaluate rehabilitation models of care, care pathways, programs, and interventions	*“I have been a leader in the development of occupational therapy in primary care”* Professor, OT (participant 102) *“My research has involved establishing community-based programs for people with aphasia in my local community… This kind of impact is difficult to capture in impact factors or other metrics but has been very meaningful to the individuals participating in these programs.”* Professor, SLP (participant 54) *“The research impact has had direct clinical implications for treating posterior shoulder instability and tightness issues.”* Instructor, PT (participant 63)
	Develop, implement and evaluate clinical tools and resources	*“I have advanced objective measurement approaches in the study of pelvic floor disorders in females, including the development of specialized tools and techniques for this purpose.”* Professor, PT (participant 45) *“Development of practical resources for clinicians”* Assistant Professor, OT (participant 46)
	Advance knowledge to influence rehabilitation clinical practice	*“Advanced knowledge of how communicative participation is experienced and impacted in people living with dysarthria”* Associate Professor, SLP (participant 6) *“Advance knowledge of post-fracture care”* Professor, PT (participant 66)
Shape rehabilitation research and practice directions	Advance research methodology	*“Advanced research reporting methods of clinical trials; advanced design and conduct of pilot and feasibility studies”* Professor, PT (participant 103)
	Set national and international research priorities	*“Invitation to serve on national and international interdisciplinary advisory boards guiding research priority setting in my field.”* Professor, OT (participant 14)
	Influence policy through research and advocacy	*“Change to provincial funding for physiotherapy in long-term care”* Assistant Professor, PT (participant 52) *“Utilization in advocacy efforts by local community organizations to improve accessibility of systems”* Professor, OT (participant 31)
	Advance conceptual frameworks in rehabilitation	*“I have contributed to a decolonial vision of rehabilitation.”* Assistant Professor, PT (participant 33)
Elevate excellence in rehabilitation education	Establish educational standards	*“My work has contributed to creating benchmarked expectations for clinical practice for entry to practice physiotherapy students”* Associate Professor, PT (participant 3)
	Advance pedagogy, curriculum and teaching innovations	*“Developed innovative sensorized manikins for simulation in teaching”* Professor, Audiology (participant 95) *“Advances in knowledge related to teaching pedagogy for physical therapy and other health disciplines learners”* Assistant Professor, PT (participant 12)
	Lead professional development	*“Continuing education workshops for clinicians in Canada and internationally”* Professor, SLP (participant 56)
Build research capacity in rehabilitation research	Mentorship of trainees	*“I am particularly proud of my 30 trainees throughout my career.”* Professor, OT (participant 80)
	Establish networks and organizations to support research and trainee development	*“Local, provincial, and national networks including… rehab research and training networks, communities of practice”* Associate Professor, PT (participant 50)
	Increase research capacity in clinical environments	*“Increased engagement of physiotherapists in acute care research”* Professor, PT (participant 103)
Knowledge mobilization and engagement in rehabilitation science research	Knowledge mobilization of research and best clinical practice to clinicians, patients and the community	*“Improving awareness and use of best practice guidelines in clinical practice”* Assistant Professor, PT (participant 12) *“Media appearances (TV, podcasts, lectures on internet)”* Professor, SLP (participant 56)
	Sustained engagement with patients and the community	*“I work deeply with communities - conducting collaborative research and many hours are spent working with communities and people”* Associate Professor, OT (participant 88)
	Disseminate research through presentations nationally and internationally	*“I have made significant efforts to disseminate this work internationally through lectures (75 invited lectures)”* Full Professor, Audiology (participant 90) *“I have given several presentations over the past 2 years…locally, provincially and nationally, to different interested parties (professional associations, regulators, health care institutions)”* Assistant Professor, OT (participant 60)

## Discussion

Using a mixed methods approach incorporating publicly available and survey data, this study provides a contemporary national characterization of research productivity and impact of 685 rehabilitation science faculty across Canada. Research environment findings highlight that key research supports and resources are established within Canadian rehabilitation science research, though their organization varies between academic units. Bibliometric and grant funding indices suggest that rehabilitation science faculty are productive researchers, for example with greater h-indices compared to research conducted approximately a decade ago [4]. Faculty descriptions of research impact highlight that impact extends beyond traditional scholarly metrics, providing novel insights into how rehabilitation science faculty conceptualize the impact of their research.

### Rehabilitation science research environment

Research environment characteristics provide important context for interpreting research productivity and impact, as factors such as research strategy, infrastructure and capacity influence research productivity.[12,47] Most rehabilitation science academic units in Canada reported having key research supports in place, including a research strategic plan, pre- and post-grant award administrative support, and access to research laboratories. Access to specific research facilities varied across academic units, with wide IQRs and a U-shaped, bimodal distribution suggesting differing approaches to research infrastructure, such as prioritizing individual or shared laboratory space, and either University campus or hospital-based research facilities. For example, a median (IQR) of 39% (11–99%) of rehabilitation science faculty had access to their own research laboratory space, with a similar pattern in PT (30% [16–80%]) and OT (30% [13–56%]) and higher access in CSD (100% [60–100%]). This variability may reflect rehabilitation science discipline-specific research approaches, institutional priorities and the predominance of clinical and health services research conducted by academic units, which can occur across academic and clinical settings. Collectively, findings suggest that key research supports and resources are established within Canadian rehabilitation science research, though their organization varies between academic units. However, only about one-third of academic units reported knowledge translation support and research labs within community organizations. Addressing these gaps may facilitate dissemination of findings to those affected by the research and partnerships that align research with community needs, supporting socially accountable research.[48]

### Bibliometric and grant funding indices of research productivity and impact

Bibliometric indices give insight into research productivity and impact, with h-index being one of the most frequently used.[3,31] Results indicate that these metrics are higher than a decade ago among rehabilitation science faculty in Canada. Median h-indices for all rehabilitation science faculty combined are 9, 19, and 32 for Assistant, Associate, and Full Professors, respectively, compared with 5, 11, and 18 reported by MacDermid et al. using data collected in 2013 for PT and OT faculty [4]. Although differences in discipline composition limit direct comparisons, discipline-specific values in the current study followed a similar pattern across ranks (Assistant, Associate, Full Professor) in PT (10, 23, 40), OT (9, 18, 26) and CSD (9, 17, 27), suggesting that greater h-indices are observed across disciplines, with the largest absolute increase among Full Professors. These findings may reflect shifts in research and publishing practices in rehabilitation science, including increasing publication volume, greater emphasis on publishing high-level evidence research (e.g., clinical trials, evidence syntheses) that may be more highly cited, and more cross-disciplinary, multi-author research teams [16,49–51]. Interpreting and using these values to inform research performance evaluations must consider variation in faculty roles within academic appointment ranks. For example, differences in time allocated to research can vary within academic rank (e.g., service roles, research chair position) which will influence research expectations and performance. While this limits direct comparisons, the contemporary bibliometric medians and interquartile ranges reported in this study offer useful reference points for research performance evaluation across academic appointments.

Research grants are also an indicator of research productivity and impact, reflecting the ability to support sustained research activity for impact to rehabilitation science and clinical practice [52,53]. In the current study, patterns of Tri-Council grant funding varied by academic appointment, with funding understandably concentrated among faculty who have research-focused appointments (Assistant, Associate, Full Professors) and more senior ranks (e.g., Full Professors). Mean funding exceeded median values, indicating a right-skewed distribution of data in which a smaller number of faculty account for a large proportion of total funding, consistent with the increasingly competitive nature of Tri-Council grant funding in Canada [53]. Tri-Council funding exceeded CIHR funding alone across all rehabilitation science disciplines, particularly in OT and CSD. For example, among Full Professors, 87% of median Tri-Council funding in PT was from CIHR, compared with 56% in OT and 0% in CSD, reflecting the variation in funding sources across rehabilitation science disciplines and breadth of rehabilitation science research that extends beyond CIHR’s health-specific mandate [16,49].

In contrast to bibliometric indices, median CIHR funding awarded as PI to all rehabilitation science faculty combined did not demonstrate comparable growth over the past decade and remains similar to values reported for rehabilitation science faculty by MacDermid et al., using data collected in 2013 on PT and OT.[4] However, discipline-specific patterns are evident when comparing with 2013 data, including greater median CIHR funding among PT faculty, particularly at senior ranks. For example, median funding for PT Full Professors in the current study was $736,252, compared with $199,259 for combined PT and OT Full Professors in 2013. In contrast, OT and CSD demonstrated similar or lower median CIHR funding relative to 2013 values, alongside evidence of greater proportion of funding from non-CIHR sources. Across all disciplines, median funding values likely underrepresent total funding received because co-investigator funding was not captured, as it is not consistently reported across Tri-Council funding databases. With the shift toward funding larger, team-based grants with more co-investigators, [54] this may have concentrated funding among a smaller number of PIs while underestimating funding for co-investigators.

A novel contribution of the present study is characterizing the research productivity and impact of faculty with teaching focused appointments. As expected, bibliometric and grant funding indices for Professors of Teaching and Instructors were lower than research focused appointments. For example, across all rehabilitation science faculty, Assistant Professors of Teaching had a median (IQR) h-index of 2 (0–5) and Tri-Council grant funding of $0 ($0–0), compared with 9 (6–16) and $0 ($0–130,050) for Assistant Professors. Lower values likely reflect differences in role expectations, including a smaller percentage of time allocated to research. Additionally, while this study did not capture the type of research conducted by individual faculty members, those in teaching focused appointments may be more engaged in the scholarship of teaching and learning, which is characterized by different citation practices, knowledge mobilization pathways and funding mechanisms compared to clinically oriented rehabilitation research.[55,56]

### Associations with research productivity and impact

For all rehabilitation science faculty, associations with both h-index and Tri-Council funding awarded were primarily driven by individual faculty characteristics rather than discipline or academic unit factors. Publication count was associated with both h-index and Tri-Council funding. While its association with h-index is expected, the finding that publication count, but not h-index, was associated with Tri-Council funding aligns with findings in other health disciplines showing stronger associations between national funding and publication count than h-index. [57] This finding may be partly a result of the measures themselves, as publication count and grant funding are both linear indices, which may be more strongly associated than a linear and non-linear index (i.e., h-index). However, it contrasts with prior research exploring associations with CIHR funding in rehabilitation science where h-index was associated with grant funding.[4] This difference may reflect variations in disciplines, analysis approaches, or potentially a reduced emphasis on citation-based metrics as indicators of research excellence.[58]

Appointment title was also associated with both h-index and Tri-Council funding, likely reflecting time allocated to research within appointments and the cumulative nature of research productivity and impact over an academic career. Although, the presence of senior rank faculty without Tri-Council funding highlights that success in Tri-Council grant funding competitions is not uniform. While median funding values are greater than zero for Full Professors, the lower bound of zero in the interquartile range indicates that at least 25% of Full Professors have no Tri-Council funding across all rehabilitation science disciplines combined, as well as within OT and CSD. While discipline was not significant in regression models, this could reflect discipline-specific funding patterns observed across data sources. For example, survey findings from Heads of academic units indicate that Tri-Council sources comprise approximately 50% of PT academic unit funding over the past five years, compared with approximately 25% in OT and CSD, where a greater proportion of funding is received from non-Tri-Council sources, such as National targeted, Provincial and Internal University funding. Senior rank faculty without Tri-Council funding may also reflect differences in institutional priorities, as not all Canadian rehabilitation science academic units are situated in research intensive universities [59].

Variability in h-index between academic units was observed, though it was not explained by measured research environment factors, suggesting that other unmeasured factors, such as institutional culture or collaboration networks, may contribute. In contrast, there was minimal between-unit variability in Tri-Council funding awarded. This could suggest that Tri-Council funding successes are more strongly driven by characteristics of individual researchers (e.g., appointment, stage of career) rather than the local research environment, but this should be interpreted cautiously given the limitations of collecting comprehensive research funding and research environment data.

### Broad conceptualization of self-described research impact

Rehabilitation science faculty descriptions highlight a broad conceptualization of research impact. While one theme captured research impact through bibliometric indices, research grant funding and awards, faculty also identified impact across rehabilitation clinical practice, education, research and practice directions, capacity building, knowledge mobilization and engagement. The breadth of this impact reflects the applied nature of rehabilitation research and aligns with principles of responsible research assessment, for example the Declaration of Research Assessment (DORA) and contemporary researcher impact frameworks [14,60]. Across these frameworks, research impact is described as encompassing contributions to knowledge, tools, methodologies; development of individuals and collaborations; strengthening the research community; and contributions to society. This comprehensive view of impact is reflected in the shift towards narrative-style academic CVs that enable researchers to describe research impact broadly [61,62]. Qualitative findings in this study highlight the multidimensional nature of research impact in rehabilitation science and support the use of broader approaches to research performance evaluations that recognize the diverse scholarly, clinical, educational, and societal contributions of rehabilitation science research.

### Strengths and limitations

This study has several strengths. The mixed methods design, incorporating publicly available and survey data, was reported in accordance with guidelines for survey and observational research, supporting comprehensive and transparent reporting. The use of standardized data extraction protocols and training procedures across research team members contribute to research rigor. However, several limitations should be acknowledged. Faculty appointment descriptions and roles vary, ranging from typical “40/40/20” mixed teaching, research and service appointments, teaching focused appointments that may have some or no time allocated to research, and dedicated research roles with 50–100% protected research time. Naming traditions, time allocated to research, role boundaries and expectations vary across time and institutions; and may not have been included in faculty appointment titles in the same way. This introduces inherent potential for error, which was mitigated by applying a standardized approach to categorizing appointment titles and through discussions with the research team, that includes long-standing leadership experience in rehabilitation science academic units internationally. Despite efforts to standardize data extracted across the Tri-Councils, substantial heterogeneity in data availability and reporting across research grant funding databases may have underestimated or misclassified research funding. Not being able to capture funding as co-investigator and from sources outside Tri-Councils provides only a partial view of overall research funding which likely underestimates funding profiles. Additionally, the number of head and faculty survey responses received were below estimated sample sizes, which may have reduced the precision of estimates and introduced response bias that may limit representativeness. Regression analyses incorporating research environment characteristics were restricted to a subset of faculty with data from Heads of academic units, which limits direct comparison between models. Furthermore, research environment variables were measured using a limited set of indicators and may not have captured important contextual factors such as collaboration networks, leadership, or institutional culture. As such, the observed lack of association between research environment characteristics and h-index should be interpreted with caution.

## Conclusion

This study provides a contemporary, national characterization of research productivity and impact across rehabilitation science faculty in Canada. Findings suggest established research environments with rehabilitation science faculty that are productive and impactful researchers, with bibliometric and grant funding indices that have increased over the past decade. Rehabilitation science faculty broadly conceptualize the impact of their research beyond traditional scholarly metrics, including impact to clinical practice, research and practice directions, education, capacity building, knowledge mobilization and engagement. Together, findings support use of bibliometric and grant funding indices as components of research performance evaluation, while emphasizing the importance of interpreting these measures in context and recognizing a broad conceptualization of research impact.

## Supporting information

S1 File STROBE, CHERRIES and CROSS reporting checklists. (PDF)

S2 File Publicly available data collection protocol. (DOCX)

S3 File Survey questions. (DOCX)

S4 File Heads of academic units, survey research environment data. (DOCX)

S5 File Comparisons of bibliometric and grant funding by appointment. (DOCX)

S6 File Regression models. (DOCX)
