# Supplementary material for: Research productivity and impact of rehabilitation science faculty across Canada: An observational study of publicly available and survey data
Source: PLoS One. 2026 Sep 15;21(9):e0357806. doi: 10.1371/journal.pone.0357806 (PMC13577373; doi:10.1371/journal.pone.0357806)
Supplement: Supplementary file 2 [file pone.0357806.s002.docx]

**Supplement 2: Publicly available data collection protocol**

**Academic unit website data**

For each full-time faculty member, use university academic website to extract:

1. Name
2. Email address
3. Discipline
4. Appointment title and stage of career (e.g., Instructor, Assistant Professor)
5. Administrative or research chair roles, as applicable

**Bibliometrics data extraction from Publish or Perish**

1. Select Google Scholar Profile Search
2. Select find a profile.
3. Input name identified from university academic website.
   a. Results will populate with profile name, affiliation, and associated labels. It should be clear which name to select.
4. Organize search results by their contribution to the h-index. To do this click the “Rank” column title.
5. Inspect all results contributing to the h-index as well as the subsequent 20. These should be inspected for author name, co-authors, institution and academic affiliations, title, abstract and research content.
   a. If needed to ensure the correct full text was inspected, use the Google Scholar Profile to find the full text article.
   b. If the full text is not available in Google Scholar Profile, search for it using our institutional library search engine (e.g., Omni).
6. If there is misattribution, remove the reference by deselecting the box in the first column. Continue to inspect 20 results after the h-index.
7. If there is a duplicate reference, the title and author names will be the same, however, it will separate the number of citations and the bibliometrics.
   a. To merge entries, drag the duplicate onto the box of the corresponding entry.
   b. Check to ensure the bibliometrics have updated.
8. To export results to excel go to the right column where it says Copy Results.
   a. Select “Metrics for Excel.”

Notes for bibliometrics data extraction:

- If an individual does not have a Google Scholar Profile, search using Google Scholar.
- Using Google Scholar searches (opposed to Google Scholar Profile) can have more improperly attributed results.
  - Using this method also requires searching using multiple searches if a person has changed their last name to get all of the entries.
  - In contrast, if an individual has changed their last name but has a Google Scholar Profile, publications for both surnames appear.

**Grant funding data extraction**

**Decisions for Tri-Council Funding**

CIHR only

SSHRC only

NSERC only

All Tri-Councils

| **Special Case** | **Decision** |
| --- | --- |
| Fellowship Awards | Not included |
| Doctoral Awards | Not included |
| Travel Awards | Not included |
| Grants before competition year 2009 | Not included |
| LOI with no attached funding | Not included |
| LOI with funding attached | Include – LOI counted separately from full grant amount |
| Grants with same project name but entered in database separately | Include both – count as separate grants |
| Grants with same project name but entered in database separately | Include both – count as separate grants  *At times it appears some may be separate grants and others are a main award with a supplemental amount. With no confirmation from NSERC, if entered separately in the database (different programs, institutions, etc.), they were counted as separate grants. This does not apply to different instalments for the same grant. |
| Grants with same project name but entered in database separately | Decision made based on information provided directly by SSHRC.   - Separate grants – counted independently - Main grant with supplemental amount – counted together |
| External Funding Partner & External Applicant Partners – NOT Tri-Council  External Funding Partners – organizations that will provide a portion of funding to a CIHR grant.  External Applicant Partners – organizations identified by the applicants themselves that contribute to research projects. There is no formal relationship between CIHR and Applicant Partners. | Funding is included and external organization is recorded. |
| External Funding Partner – Joint Tri-Council  Note: partnerships are almost always between CIHR and NSERC, and the grant will always appear in CIHR due to multiple PIs allowed to be listed where SSHRC and NSERC are restricted to one. | Tri-council partnered funding (joint) is accounted for in each council separately (i.e., CIHR amount is counted as CIHR funding only, NSERC amount is counted as NSERC funding only). Partnership is recorded.  Important: CIHR allows for multiple PIs where NSERC only allows for 1 project lead name. Therefore, if the person is not the selected project lead name (NSERC) only the CIHR amount is captured. NSERC amount may be captured under another Project Lead Name (if another faculty member), or it may not be captured at all (i.e., PLN is not within PT, OT, or CSD faculty).. |

**Procedures for searching each database**

**CIHR**

For CIHR, the number of grants and amount as Principal Investigator will be extracted. This must be searched as “All Results” or else not all grants will appear.

1. Search using the format: First name Last name (no comma).
   a. Comma indicates “or” where a space specifies “and.”
2. The default for the category should be “All Results.”
   a. Do not change this category unless the name is extremely common and the results are too demanding to be manually inspected (e.g., Sue Peters retrieves 641 results).
3. Inspect name, Principal Investigator, Co-Investigators, Supervisors, Research Institution, Department, Program, and Abstract.
   a. A common issue in this database is that a grant is retrieved from a search due to a shared first or last name as a different researcher. These entries are not included.
4. Do not include any improperly attributed grants.
5. Use Excel to add the monetary amount.
6. After every search, it is imperative you use the left column to remove filters. The database is very sensitive if this column is not cleared, and results will not populate properly.
7. If an individual has changed their last name, the protocol needs to be repeated for both last names and the results must be merged.

**Important:** In CIHR, all installments will appear for a grant in the same entry. This allows the number of results in CIHR to be recorded as the number of grants.

**NSERC**

For NSERC, only data as project lead can be extracted

1. Search using the fiscal year.
2. Search using the format Last Name, First Name.
3. Inspect Project Lead Name, department, institution, program, and research subject.
4. Use Excel to add the monetary amount.
5. Record the amount of funding received and the number of grants.
6. If an individual has changed their last name, the protocol needs to be repeated for both last names and the results must be merged.

**Important:** In NSERC, the same grant will be listed for each fiscal year an installment was given. The project lead name, competition year, institution, program, etc. will remain the same. The fiscal year and the installment number will change. Record grants with the same project name one time.

**SSHRC**

For SSHRC, only data as applicant can be extracted.

Last Name, First Name.

1. Search using the fiscal year.
2. Search only using faculty’s surname.
   a. If both first name and last name are used, often results would display either no results or the incorrect number of results.
   b. For those with surnames made up of two separate last names (e.g., Debbie Laliberte Rudman), search both surnames. The 2 last names cannot be searched together, or it will result in an error code. Searching either surname usually yields the same results.
3. Navigate to the surname using the arrows.
   a. Once you navigate to their name all the results will appear here. It may appear as a great number of results to search through initially due to the page number, however their results will all be organized together.
4. Use Excel to add the monetary amount.
5. Record the amount of funding received and the number of grants.
6. If an individual has changed their last name, the protocol needs to be repeated for both last names and the results must be merged.
7. Once all grant funding received from SSHRC data was extracted for PT, OT and CSD faculty, a list was compiled and sent to SSHRC, who provided the amount awarded for each grant.

**Important:** In SSHRC, the same grant will be listed for each fiscal year an installment was given. The applicant, competition year, area of research will remain the same. It is the fiscal year that will be changed with each entry. Record grants with the same project name one time.
