# Supplementary material for: Research productivity and impact of rehabilitation science faculty across Canada: An observational study of publicly available and survey data
Source: PLoS One. 2026 Sep 15;21(9):e0357806. doi: 10.1371/journal.pone.0357806 (PMC13577373; doi:10.1371/journal.pone.0357806)
Supplement: Supplementary file 3 [file pone.0357806.s003.docx]

**Research productivity and impact of rehabilitation science faculty across Canada:**

**Heads of Units / Schools survey**

**1. Heads of Units / Schools survey - Profile of faculty**

1. How many faculty are employed in the Unit / School you lead?
   a. Open text box
2. How many tenure track faculty are employed in the Unit / School you lead?
   a. Open text box
3. Of the tenure track faculty, how many are:
   a. Assistant professors (open text box)
   b. Associate professors (open text box)
   c. Full professors (open text box)
4. How many full-time faculty in the Unit / School you lead have:
   a. Designated research time ≥60% or are research chairs? (open text box)
   b. Typical academic roles with 40% research assignments? (open text box)
   c. Full-time teaching-focused roles with ≤10% research assignments? (open text box)
   d. Other (open text box x2)
5. How many part-time teaching faculty are employed in the Unit / School you lead?
   a. Open text box
6. How many professors emeriti are in the Unit / School you lead?
   a. Open text box
7. Is the information about faculty listed on the website of the Unit / School you lead accurate?
   a. Yes
   b. No
8. Please feel free to elaborate on any of the questions above to provide further context / information.
   a. Open text box

**2. Heads of Units / Schools survey - Research support**

1. For the Unit / School you lead, which of the following are in place to support research activity? Please select all that apply.
   a. Research strategic plan
   b. Pre-award grant support
   c. Post-award grant support
   d. Knowledge translation support
   e. Research laboratories within Unit / School
   f. Research laboratories within Hospital
   g. Research laboratories within community organization
   h. Other: Please briefly describe
2. Please feel free to elaborate on any of the questions above to provide further context / information.
   a. Open text box

**3. Heads of Units / Schools program survey - Research facilities**

1. Do the research faculty in the Unit / School you lead have the research facilities (e.g., laboratories) they need to conduct their research?
   a. Yes
   b. No
2. What percentage of faculty in the Unit / School you lead:
   a. Have their own research lab space? (open text box)
   b. Have access to a shared research lab? (open text box)
   c. Do their research in an affiliated hospital? (open text box)
   d. Work in collaboration with community organization? (open text box)
3. What would you estimate as the distribution of type of research done by faculty in the Unit / School you lead? (Please add to 100%)
   a. Basic Science
   b. Clinical research
   c. Social science research
   d. Applied development / design research
   e. Community health / Public health research
   f. Health services research
   g. Population health
   h. Economic evaluation
   i. Other, please briefly describe
4. Please feel free to elaborate on any of the questions above to provide further context / information.
   a. Open text box

**4. Heads of Units / Schools survey - Research funding**

1. What is the total amount of research funding received over the past 5 years in the Unit / School you lead?
   a. Open text box
2. What percentage of the total amount of research funding received over the past 5 years in the Unit / School you lead was funded by the following sources? (Please add to 100%)
   a. CIHR (or equivalent in another country)
   b. NSERC (or equivalent in another country)
   c. SSHRC (or equivalent in another country)
   d. Provincial agencies (or equivalent in another country)
   e. National Targeted Funding (Including non-profit / charity funding, e.g., Heart and Stroke, Arthritis Society, Mental Health)
   f. Industry
   g. Professional Organization
   h. Local Hospital or Municipal Funding
   i. Internal University Academic Funds
3. Please feel free to elaborate on any of the questions above to provide further context / information.
a. Open text box

**5. Heads of Units / Schools survey - Research trainees**

1. How many of the following trainees are currently supervised by faculty in the Unit / School you lead, inclusive of trainees enrolled outside the Unit / School?
   a. MSc students (open text box)
   b. PhD students (open text box)
   c. Postdoctoral researchers (open text box)
4. Please feel free to elaborate on any of the questions above to provide further context / information.
b. Open text box

**Heads of Units / Schools survey – University information**

If you feel comfortable, we invite you to provide the name of the University where you are the Head and your discipline.

By providing this information, we will be able to pair your research environment survey responses with bibliometric, Tri-Council grant funding and faculty survey data characterizing research productivity and impact.

The University in which you work will be tagged as an identifier to enable de-identified data to be downloaded. After pairing data, all data will be de-identified, study results will be presented in aggregate and no individual participant will be identifiable. Survey responses will be kept confidential and secure within our research team.

1. Name of the University where you are the Head: ______________________
2. Please select your discipline:
   a. Physical Therapy
   b. Occupational Therapy
   c. Communication Sciences and Disorders
      - Please select the program(s) within the Communication Sciences and Disorders School / Unit you lead
        - Speech and Language Pathology
        - Audiology
3. I prefer to not disclose. If you feel comfortable, please let us know why: _____________

Thank you for completing this survey.

If you have any questions about this research study, please feel free to contact one of our team members.

| **Alison Rushton**  Joint Principal Investigator  Director and Professor  School of Physical Therapy  arushto3@uwo.ca | **Joy MacDermid**  Joint Principal Investigator Distinguished University Professor  School of Physical Therapy  jmacderm@uwo.ca | **Lisa Archibald**  Co-Investigator  Director and Professor  School of Communication Sciences and Disorders  larchiba@uwo.ca |
| --- | --- | --- |
| **Lynn Shaw**  Co-Investigator  Director and Professor  School of Occupational Therapy  leshaw@uwo.ca | **Christine Meston**  Co-Investigator  Assistant Professor  Audiology, School of Communication Sciences and Disorders  cmeston@uwo.ca | **Allyson Page**  Co-Investigator  Associate Professor  Speech Language Pathology, School of Communication Sciences and Disorders  [allyson.page@uwo.ca](mailto:allyson.page@uwo.ca) |
| **Katie Kowalski**  Co-Investigator  Postdoctoral Research Associate  School of Physical Therapy  kkowals7@uwo.ca |  |  |

**Research productivity and impact of rehabilitation science faculty across Canada:**

**Faculty survey**

**Section 4. Faculty survey - Research impact**

We know bibliometric and grant funding indices provide a limited view of research impact. Research impact may also include contributions, effects, changes or advantages that research brings to the economy, society, culture, policy, clinical practice, health, the environment and / or quality of life.

In the question below, you may consider research to be broadly defined, for example including the scholarship of discovery (e.g., advance knowledge), integration (e.g., synthesize information), application (e.g., application of disciplinary expertise), and / or teaching and learning (e.g., systematic study of teaching and learning processes).

1. Please describe or list the impact of your program of rehabilitation science research.
   a. (open text box)
