# Supplementary material for: Research productivity and impact of rehabilitation science faculty across Canada: An observational study of publicly available and survey data
Source: PLoS One. 2026 Sep 15;21(9):e0357806. doi: 10.1371/journal.pone.0357806 (PMC13577373; doi:10.1371/journal.pone.0357806)
Supplement: Supplementary file 4 [file pone.0357806.s004.docx]

**Supplement 4 – Heads of academic units research environment survey data**

**Profile of faculty**

Table 1. Number of faculty reported by Heads of academic units in the unit they lead

| **Faculty** | **All disciplines**  **(n=35)** | **PT**  **(n=15)** | **OT**  **(n=11)** | **CSD**  **(n=7)** |
| --- | --- | --- | --- | --- |
| ≥60% research / CRC  Total number (n)  Median (IQR)  Mean (SD) | 115  2 (1-6)  3 (3) | 66  3 (2-7)  4 (4) | 37  3 (10-15)  3 (1-5) | 6  1 (0-1)  1 (1) |
| 40% research  Total number (n)  Median (IQR)  Mean (SD) | 267  7 (5-9)  8 (7) | 116  7 (5-8)  8 (9) | 88  9 (5-10)  8 (5) | 49  7 (6-9)  7 (2) |
| ≤10% research  Total number (n)  Median (IQR)  Mean (SD) | 122  3 (1-6)  4 (3) | 58  4 (2-6)  4 (3) | 42  4 (1-7)  4 (3) | 18  2 (0-6)  3 (4) |
| Other % research  Total number (n)  Median (IQR)  Mean (SD) | 52  0 (0-3)  1 (2) | 14  0 (0-1)  1 (2) | 19  0 (0-3)  2 (3) | 19  2 (1-5)  3 (3) |
| Part-time  Total number (n)  Median (IQR)  Mean (SD) | 183  5 (0–8)  5 (5) | 90  4 (1-9)  6 (6) | 57  5 (2-8)  6 (5) | 30  1 (0-9)  4 (5) |
| Emerti  Total number (n)  Median (IQR)  Mean (SD) | 94  3 (2-4)  3 (2) | 42  3 (2-4)  3 (2) | 31  3 (2-4)  3 (2) | 17  3 (1-4)  2 (2) |

All disciplines include 2 academic units with unknown discipline, which are not included in discipline specific columns. n= represents the number of survey responses received from Heads of academic units.

Total number represents the sum of the number of faculty. Median and mean reflect numbers per unit.

Other percentage research included <10%, 20-30% and 50-55% with descriptions of faculty with greater service responsibilities and contract academic staff.

CRC: Canada Research Chair, PT: Physical Therapy, OT: Occupational Therapy, CSD: Communication Sciences and Disorders

**Research support**

Table 2. Mean percentage of academic units reporting research supports are in place.

| **Research supports** | **All disciplines**  **(n=35)** | **PT**  **(n=15)** | **OT**  **(n=11)** | **CSD**  **(n=7)** |
| --- | --- | --- | --- | --- |
| Research strategic plan (%) | 60 | 73 | 46 | 57 |
| Pre-award grant support (%) | 97 | 100 | 100 | 86 |
| Post-award grant support (%) | 69 | 80 | 64 | 57 |
| Knowledge translation support (%) | 37 | 53 | 27 | 14 |
| Research labs within Unit (%) | 83 | 87 | 82 | 86 |
| Research labs within Hospital (%) | 71 | 80 | 64 | 57 |
| Research labs within community organization (%) | 34 | 33 | 55 | 0 |
| Other (%) | 29 | 40 | 27 | 0 |

All disciplines include 2 academic units with unknown discipline, which are not included in discipline specific columns. n= represents the number of survey responses received from Heads of academic units.

Other research supports included university research institutes and centers, research labs within the university, research mentorship, and teaching buyout to increase research time.

**Research facilities**

Mean percentage of academic units reporting that faculty have the research facilities (e.g., laboratories) they need to conduct their research. n= represents the number of survey responses received from Heads of academic units, with n=1 unknown discipline.

- All disciplines (n=32): 97%
- PT (n=14): 100%
- OT (n=10): 90%
- CSD (n=7): 100%

Table 3. Percentage of academic units reporting access to research facilities.

| **Research facilities** | **All disciplines**  **(n=34)** | **PT**  **(n=15)** | **OT**  **(n=11)** | **CSD**  **(n=7)** |
| --- | --- | --- | --- | --- |
| Own research lab space (%)  Median (IQR)  Mean (SD) | 39 (11-99)  48 (39) | 30 (16-80)  45 (39) | 30 (13-56)  40 (35) | 100 (60-100)  75 (37) |
| Access to a shared research lab (%)  Median (IQR)  Mean (SD) | 26 (4-98)  42 (41) | 27 (11-100)  52 (44) | 50 (3-95)  50 (43) | 2 (0-18)  14 (23) |
| Do research in an affiliated hospital (%)  Median (IQR)  Mean (SD) | 22 (8-40)  30 (31) | 27 (16-60)  39 (33) | 10 (5-40)  30 (35) | 10 (5-20)  12 (10) |
| Work in collaboration with community organizations (%)  Median (IQR)  Mean (SD) | 31 (11-82)  43 (37) | 45 (23-92)  53 (39) | 25 (11-78)  41 (38) | 20 (1-31)  26 (35) |

All disciplines include 1 academic unit with unknown discipline, which is not included in discipline specific columns. n= represents the number of survey responses received from Heads of academic units.

**Research type**

Table 4. Percentage of research type conducted by rehabilitation research faculty across Canada

| **Research type** | **All disciplines**  **(n=33)** | **PT**  **(n=15)** | **OT**  **(n=10)** | **CSD**  **(n=7)** |
| --- | --- | --- | --- | --- |
| Basic science (%)  Median (IQR)  Mean (SD) | 10 (0-15)  10 (12) | 10 (2-16)  12 (12) | 0 (0-9)  4 (6) | 15 (6-20)  17 (17) |
| Clinical research (%)  Median (IQR)  Mean (SD) | 40 (30-60)  46 (23) | 49 (35-68)  49 (22) | 29 (14-40)  29 (16) | 60 (50-78)  63 (18) |
| Social science research (%)  Median (IQR)  Mean (SD) | 3 (0-20)  11 (16) | 0 (0-4)  4 (7) | 21 (13-40)  26 (19) | 0 (0-5)  4 (8) |
| Applied development/design research (%)  Median (IQR)  Mean (SD) | 0 (0-5)  5 (10) | 0 (0-5)  6 (11) | 0 (0-10)  7 (13) | 0 (0-3)  2 (4) |
| Community/public health research (%)  Median (IQR)  Mean (SD) | 5 (0-13)  9 (10) | 5 (4-16)  10 (10) | 11 (9-15)  13 (10) | 0 (0-3)  2 (4) |
| Health services research (%)  Median (IQR)  Mean (SD) | 10 (4-20)  15 (17) | 14 (5-23)  16 (14) | 9 (1-18)  16 (25) | 10 (0-13)  9 (11) |
| Population health (%)  Median (IQR)  Mean (SD) | 0 (0-4)  2 (4) | 0 (0-3)  1 (2) | 3 (0-9)  4 (5) | 0 (0-0)  1 (4) |
| Economic evaluation (%)  Median (IQR)  Mean (SD) | 0 (0-0)  1 (3) | 0 (0-2)  2 (4) | 0 (0-0)  0 (0) | 0 (0-0)  0 (0) |
| Other (%)  Median (IQR)  Mean (SD) | 0 (0-0)  2 (5) | 0 (0-1)  2 (5) | 0 (0-0)  2 (3) | 0 (0-0)  1 (2) |

All disciplines include 1 academic unit with unknown discipline, which is not included in discipline specific columns. n= represents the number of survey responses received from Heads of academic units.

Other research types included educational research, Indigenous community-led research, and epidemiology.

**Research funding**

Table 5. Percentage of research funding sources received in the last 5 years

| **Research funding sources** | **All disciplines**  **(n=24)** | **PT**  **(n=12)** | **OT**  **(n=7)** | **CSD**  **(n=5)** |
| --- | --- | --- | --- | --- |
| CIHR or equivalent in another country (%)  Median (IQR)  Mean (SD) | 31 (11-48)  32 (23) | 42 (30-50)  41 (20) | 24 (16-41)  30 (23) | 5 (1-6)  12 (21) |
| NSERC or equivalent in another country (%)  Median (IQR)  Mean (SD) | 0 (0-3)  3 (5) | 1 (0-3)  2 (2) | 0 (0-1)  1 (1) | 7 (0-7)  7 (9) |
| SSHRC or equivalent in another country (%)  Median (IQR)  Mean (SD) | 6 (1-10)  10 (13) | 1 (0-2)  4 (7) | 7 (6-19)  14 (14) | 11 (10-15)  17 (16) |
| Provincial agencies or equivalent in another country (%)  Median (IQR)  Mean (SD) | 8 (4-13)  12 (13) | 9 (5-11)  12 (13) | 5 (3-9)  9 (12) | 12 (0-23)  15 (16) |
| National targeted funding, including non-profit / charity funding (%)  Median (IQR)  Mean (SD) | 16 (7-28)  19 (14) | 20 (11-24)  18 (10) | 32 (9-37)  26 (18) | 4 (0-10)  9 (14) |
| Industry (%)  Median (IQR)  Mean (SD) | 0 (0-2)  2 (3) | 0 (0-2)  2 (3) | 0 (0-4)  2 (4) | 0 (0-1)  1 (1) |
| Professional organization (%)  Median (IQR)  Mean (SD) | 0 (0-1)  1 (2) | 0 (0-1)  2 (3) | 0 (0-0)  0 (0) | 0 (0-1)  1 (1) |
| Local hospital or municipal funding (%)  Median (IQR)  Mean (SD) | 3 (0-5)  3 (3) | 4 (2-5)  4 (3) | 3 (0-4)  2 (2) | 0 (0-1)  1 (2) |
| Internal University academic funds (%)  Median (IQR)  Mean (SD) | 6 (3-15)  12 (16) | 6 (4-13)  9 (8) | 5 (2-11)  12 (20) | 17 (5-21)  21 (24) |
| Other (%)  Median (IQR)  Mean (SD) | 0 (0-7)  8 (15) | 0 (0-4)  7 (16) | 1 (0-6)  3 (4) | 0 (0-35)  16 (23) |

n= represents the number of survey responses received from Heads of academic units.

Other funding sources included donations, confidential sources, foreign governments, Canadian subgrants, Canada Research Chairs, infrastructure funding, and research institutes, centers and networks.

The total amount of research funding received over the last 5 years is:

- All disciplines (n=22)
  - Median (IQR): $6,843,668 (3,241,717 – 19,252,619)
  - Mean (SD): $19,544,317 (31,166,128)
- PT (n=11)
  - Median (IQR): $15,188,246 (6,843,668 – 29,079,719)
  - Mean (SD): $21,568,450 (21,487,250)
- OT (n=6)
  - Median (IQR): $4,456,453 (2,639,003 – 6,440,642)
  - Mean (SD): $25,640,470 (53,965,150)
- CSD (n=5)
  - Median (IQR): $3,492,178 (3,158,230 – 11,793,992)
  - Mean (SD): $7,775,841 (8,048,889)

**Research trainees**

Table 6. Number of research trainees supervised by rehabilitation science faculty across Canada

| **Trainees** | **All disciplines**  **(n=30)** | **PT**  **(n=13)** | **OT**  **(n=11)** | **CSD**  **(n=6)** |
| --- | --- | --- | --- | --- |
| MSc students  Total number (n)  Median (IQR)  Mean (SD) | 517  13 (8-17)  17 (18) | 286  15 (8-27)  22 (19) | 188  13 (10-15)  17 (20) | 43  9 (5-9)  7 (3) |
| PhD students  Total number (n)  Median (IQR)  Mean (SD) | 620  18 (12-26)  21 (14) | 296  15 (13-24)  23 (17) | 229  18 (16-28)  21 (10) | 95  16 (9-21)  16 (12) |
| Postdocs  Total number (n)  Median (IQR)  Mean (SD) | 147  4 (2-6)  5 (5) | 73  5 (2-7)  6 (5) | 63  5 (3-7)  6 (5) | 11  2 (1-3)  2 (1) |
| Total trainees  Total number (n)  Median (IQR)  Mean (SD) | 1284  37 (25-50)  43 (24) | 655  38 (28-77)  50 (29) | 480  42 (33-50)  44 (18) | 149  23 (16-30)  25 (14) |

n= represents the number of survey responses received from Heads of academic units. Total number represents the sum of the number of trainees across all units. Median and mean reflect numbers per unit.
