# Supplementary material for: Research productivity and impact of rehabilitation science faculty across Canada: An observational study of publicly available and survey data
Source: PLoS One. 2026 Sep 15;21(9):e0357806. doi: 10.1371/journal.pone.0357806 (PMC13577373; doi:10.1371/journal.pone.0357806)
Supplement: Supplementary file 5 [file pone.0357806.s005.docx]

**Supplement 5 – Comparisons of bibliometric and grant funding indices by appointment title**

**All rehabilitation science faculty combined: Publication count**

The overall Kruskal-Wallis model for publication count was significant:

| **Publication count** | **Sample size** | **df** | **Test statistic** | **p** |
| --- | --- | --- | --- | --- |
| Appointment title | 685 | 6 | 401.2 | <0.001 |

Post-hoc analysis identified the following differences:

| **Dunn’s pairwise comparison of appointment titles** | | **Mean rank pairwise difference** | **Std. Error** | **Standardized Test Statistic** | **Adj. Sig.** |
| --- | --- | --- | --- | --- | --- |
| **Appointment 1 (Mean rank)** | **Appointment 2 (Mean rank)** |  |  |  |  |
| Instructor (92.7) | Assistant Professor, Teaching (136.3) | -43.6 | 36.8 | -1.2 | 1.000 |
| Instructor (92.7) | Associate Professor, Teaching (147.3) | -54.6 | 44.2 | -1.2 | 1.000 |
| Instructor (92.7) | Full Professor, Teaching (156.3) | -63.5 | 58.8 | -1.1 | 1.000 |
| Instructor (92.7) | Assistant Professor (290.7) | -198.0 | 26.8 | -7.4 | 0.000 |
| Instructor (92.7) | Associate Professor (417.3) | -324.5 | 25.7 | -12.6 | 0.000 |
| Instructor (92.7) | Full Professor (513.0) | -420.2 | 25.5 | -16.5 | 0.000 |
| Assistant Professor, Teaching (136.3) | Associate Professor, Teaching (147.3) | -11.0 | 49.2 | -0.2 | 1.000 |
| Assistant Professor, Teaching (136.3) | Full Professor, Teaching (156.3) | -20.0 | 62.6 | -0.3 | 1.000 |
| Assistant Professor, Teaching (136.3) | Assistant Professor (290.7) | -154.4 | 34.4 | -4.5 | 0.000 |
| Assistant Professor, Teaching (136.3) | Associate Professor (417.3) | -281.0 | 33.6 | -8.4 | 0.000 |
| Assistant Professor, Teaching (136.3) | Full Professor (513.0) | -376.7 | 33.4 | -11.3 | 0.000 |
| Associate Professor, Teaching (147.3) | Full Professor, Teaching (156.3) | -9.0 | 67.2 | -0.1 | 1.000 |
| Associate Professor, Teaching (147.3) | Assistant Professor (290.7) | -143.4 | 42.2 | -3.4 | 0.014 |
| Associate Professor, Teaching (147.3) | Associate Professor (417.3) | -270.0 | 41.5 | -6.5 | 0.000 |
| Associate Professor, Teaching (147.3) | Full Professor (513.0) | -365.7 | 41.4 | -8.8 | 0.000 |
| Full Professor, Teaching (156.3) | Assistant Professor (290.7) | -134.4 | 57.3 | -2.3 | 0.399 |
| Full Professor, Teaching (156.3) | Associate Professor (417.3) | -261.0 | 56.8 | -4.6 | 0.000 |
| Full Professor, Teaching (156.3) | Full Professor | -356.7 | 56.7 | -6.3 | 0.000 |
| Assistant Professor (290.7) | Associate Professor (417.3) | -126.6 | 22.1 | -5.7 | 0.000 |
| Assistant Professor (290.7) | Full Professor (513.0) | -222.3 | 21.9 | -10.2 | 0.000 |
| Associate Professor (417.3) | Full Professor (513.0) | -95.7 | 20.5 | -4.7 | 0.000 |

- Full professors had higher median publication count than:
  - Assistant professors
  - Associate professors
  - Assistant professors, Teaching
  - Associate professors, Teaching
  - Full professors, Teaching
  - Instructors
- Associate professors had higher publication count than:
  - Assistant professors
  - Assistant professors, Teaching
  - Associate professors, Teaching
  - Full professors, Teaching
  - Instructors
- Assistant professors had higher publication count than:
  - Assistant professors, Teaching
  - Associate professors, Teaching
  - Instructors
- Assistant professors had a similar publication count to Full Professors of Teaching
- Publication count did not differ between Professors of Teaching at all ranks (Full, Associate, Assistant) and Instructors

**All rehabilitation science faculty combined: h-index**

The overall Kruskal-Wallis model for h-index was significant:

| **h-index** | **Sample size** | **df** | **Test statistic** | **p** |
| --- | --- | --- | --- | --- |
| Appointment title | 685 | 6 | 421.0 | <0.001 |

Post-hoc analysis identified the following differences:

| **Dunn’s pairwise comparison of appointment titles** | | **Mean rank pairwise difference** | **Std. Error** | **Standardized Test Statistic** | **Adj. Sig.** |
| --- | --- | --- | --- | --- | --- |
| **Appointment 1 (Mean rank)** | **Appointment 2 (Mean rank)** |  |  |  |  |
| Instructor (91.5) | Assistant Professor, Teaching (120.6) | -29.0 | 36.8 | -0.8 | 1.000 |
| Instructor (91.5) | Associate Professor, Teaching (159.8) | -68.3 | 44.1 | -1.5 | 1.000 |
| Instructor (91.5) | Full Professor, Teaching (171.8) | -80.3 | 58.8 | -1.4 | 1.000 |
| Instructor (91.5) | Assistant Professor (282.3) | -190.8 | 26.8 | -7.1 | 0.000 |
| Instructor (91.5) | Associate Professor (418.0) | -326.4 | 25.7 | -12.7 | 0.000 |
| Instructor (91.5) | Full Professor (520.0) | -428.4 | 25.5 | -16.8 | 0.000 |
| Assistant Professor, Teaching (120.6) | Associate Professor, Teaching (159.8) | -39.2 | 49.1 | -0.8 | 1.000 |
| Assistant Professor, Teaching (120.6) | Full Professor, Teaching (171.8) | -51.3 | 62.6 | -0.8 | 1.000 |
| Assistant Professor, Teaching (120.6) | Assistant Professor (282.3) | -161.8 | 34.4 | -4.7 | 0.000 |
| Assistant Professor, Teaching (120.6) | Associate Professor (418.0) | -297.4 | 33.6 | -8.9 | 0.000 |
| Assistant Professor, Teaching (120.6) | Full Professor (520.0) | -399.4 | 33.4 | -12.0 | 0.000 |
| Associate Professor, Teaching (159.8) | Full Professor, Teaching (171.8) | -12.0 | 67.2 | -0.2 | 1.000 |
| Associate Professor, Teaching (159.8) | Assistant Professor (282.3) | -122.5 | 42.2 | -2.9 | 0.077 |
| Associate Professor, Teaching (159.8) | Associate Professor (418.0) | -258.2 | 41.5 | -6.2 | 0.000 |
| Associate Professor, Teaching (159.8) | Full Professor (520.0) | -360.2 | 41.3 | -8.7 | 0.000 |
| Full Professor, Teaching (171.8) | Assistant Professor (282.3) | -110.5 | 57.3 | -1.9 | 1.000 |
| Full Professor, Teaching (171.8) | Associate Professor (418.0) | -246.1 | 56.8 | -4.3 | 0.000 |
| Full Professor, Teaching (171.8) | Full Professor (520.0) | -348.1 | 56.7 | -6.1 | 0.000 |
| Assistant Professor (282.3) | Associate Professor (418.0) | -135.6 | 22.1 | -6.1 | 0.000 |
| Assistant Professor (282.3) | Full Professor (520.0) | -237.6 | 21.9 | -10.9 | 0.000 |
| Associate Professor (418.0) | Full Professor (520.0) | -102.0 | 20.5 | -5.0 | 0.000 |

- Full professors had higher median h-index than:
  - Assistant professors
  - Associate professors
  - Assistant professors, Teaching
  - Associate professors, Teaching
  - Full professors, Teaching
  - Instructors
- Associate professors had higher h-index than:
  - Assistant professors
  - Assistant professors, Teaching
  - Associate professors, Teaching
  - Full professors, Teaching
  - Instructors
- Assistant professors had a higher h-index than:
  - Assistant professors, Teaching
  - Instructors
- Assistant professors had a similar h-index as:
  - Associate professors, Teaching
  - Full professors, Teaching
- H-index did not differ between Professors of Teaching at all ranks (Full, Associate, Assistant) and Instructors

**All rehabilitation science faculty combined: Number of Tri-Council grants awarded as PI (CIHR), Project Lead (NSERC), Applicant (SSHRC)**

The overall Kruskal-Wallis model for number of Tri-Council grants awarded was significant:

| **No. Tri-Council grants** | **Sample size** | **df** | **Test statistic** | **p** |
| --- | --- | --- | --- | --- |
| Appointment title | 685 | 6 | 219.7 | <0.001 |

Post-hoc analysis identified the following differences:

| **Dunn’s pairwise comparison of appointment titles** | | **Mean rank pairwise difference** | **Std. Error** | **Standardized Test Statistic** | **Adj. Sig.** |
| --- | --- | --- | --- | --- | --- |
| **Appointment 1 (Mean rank)** | **Appointment 2 (Mean rank)** |  |  |  |  |
| Full Professor, Teaching (181.0) | Instructor (187.1) | 6.1 | 54.2 | 0.1 | 1.000 |
| Full Professor, Teaching (181.0) | Assistant Professor, Teaching (189.4) | 8.4 | 57.7 | 0.1 | 1.000 |
| Full Professor, Teaching (181.0) | Associate Professor, Teaching (192.3) | 11.9 | 62.0 | 0.2 | 1.000 |
| Full Professor, Teaching (181.0) | Assistant Professor (304.6) | -123.6 | 52.8 | -2.3 | 0.406 |
| Full Professor, Teaching (181.0) | Associate Professor (399.7) | -218.7 | 52.4 | -4.2 | 0.001 |
| Full Professor, Teaching (181.0) | Full Professor (455.9) | -274.9 | 52.3 | -5.3 | 0.000 |
| Instructor (187.1) | Assistant Professor, Teaching (189.4) | -2.3 | 33.9 | -0.1 | 1.000 |
| Instructor (187.1) | Associate Professor, Teaching (192.3) | -5.7 | 40.7 | -0.1 | 1.000 |
| Instructor (187.1) | Assistant Professor (304.6) | -117.5 | 24.7 | -4.8 | 0.000 |
| Instructor (187.1) | Associate Professor (399.7) | -212.6 | 23.7 | -9.0 | 0.000 |
| Instructor (187.1) | Full Professor (455.9) | -268.7 | 23.5 | -11.4 | 0.000 |
| Assistant Professor, Teaching (189.4) | Associate Professor, Teaching (192.3) | -3.5 | 45.3 | -0.1 | 1.000 |
| Assistant Professor, Teaching (189.4) | Assistant Professor (304.6) | -115.2 | 31.7 | -3.6 | 0.006 |
| Assistant Professor, Teaching (189.4) | Associate Professor (399.7) | -210.3 | 30.9 | -6.8 | 0.000 |
| Assistant Professor, Teaching (189.4) | Full Professor (455.9) | -266.5 | 30.8 | -8.7 | 0.000 |
| Associate Professor, Teaching (192.3) | Assistant Professor (304.6) | -111.7 | 38.9 | -2.9 | 0.085 |
| Associate Professor, Teaching (192.3) | Associate Professor (399.7) | -206.8 | 38.2 | -5.4 | 0.000 |
| Associate Professor, Teaching (192.3) | Full Professor (455.9) | -263.0 | 38.1 | -6.9 | 0.000 |
| Assistant Professor (304.6) | Associate Professor (399.7) | -95.1 | 20.4 | -4.7 | 0.000 |
| Assistant Professor (304.6) | Full Professor (455.9) | -151.3 | 20.2 | -7.5 | 0.000 |
| Associate Professor (399.7) | Full Professor (455.9) | -56.2 | 18.9 | -3.0 | 0.062 |

- Full professors were awarded a higher number of Tri-Council grants than:
  - Assistant professors
  - Assistant professors, Teaching
  - Associate professors, Teaching
  - Full professors, Teaching
  - Instructors
- Full professors were awarded a similar number of Tri-Council grants as Associate Professors
- Associate professors were awarded a higher number of Tri-Council grants than:
  - Assistant professors
  - Assistant professors, Teaching
  - Associate professors, Teaching
  - Full professors, Teaching
  - Instructors
- Assistant Professors were awarded a higher number of Tri-Council grants than:
  - Instructors
  - Assistant professors, Teaching
- Assistant Professors were awarded a similar number of Tri-Council grants as:
  - Associate professors, Teaching
  - Full professors, Teaching
- Number of Tri-Council grants awarded did not differ between Professors of Teaching at all ranks (Full, Associate, Assistant) and Instructors

**All rehabilitation science faculty combined: Tri-Council grant funding awarded as PI (CIHR), Project Lead (NSERC), Applicant (SSHRC)**

The overall Kruskal-Wallis model for Tri-Council grant funding awarded was significant:

| **Tri-Council grant funding** | **Sample size** | **df** | **Test statistic** | **p** |
| --- | --- | --- | --- | --- |
| Appointment title | 685 | 6 | 212.5 | <0.001 |

Post-hoc analysis identified the following differences:

| **Dunn’s pairwise comparison of appointment titles** | | **Mean rank pairwise difference** | **Std. Error** | **Standardized Test Statistic** | **Adj. Sig.** |
| --- | --- | --- | --- | --- | --- |
| **Appointment 1 (Mean rank)** | **Appointment 2 (Mean rank)** |  |  |  |  |
| Full Professor, Teaching (181.0) | Instructor (186.3) | 5.3 | 54.3 | 0.1 | 1.000 |
| Full Professor, Teaching (181.0) | Assistant Professor, Teaching (189.6) | 8.6 | 57.9 | 0.1 | 1.000 |
| Full Professor, Teaching (181.0) | Associate Professor, Teaching (194.5) | 13.5 | 62.1 | 0.2 | 1.000 |
| Full Professor, Teaching (181.0) | Assistant Professor (308.9) | -127.9 | 53.0 | -2.4 | 0.330 |
| Full Professor, Teaching (181.0) | Associate Professor (401.5) | -220.5 | 52.5 | -4.2 | 0.001 |
| Full Professor, Teaching (181.0) | Full Professor (451.0) | -270.0 | 52.4 | -5.2 | 0.000 |
| Instructor (186.3) | Assistant Professor, Teaching (189.6) | -3.4 | 34.0 | -0.1 | 1.000 |
| Instructor (186.3) | Associate Professor, Teaching (194.5) | -8.2 | 40.8 | -0.2 | 1.000 |
| Instructor (186.3) | Assistant Professor (308.9) | -122.7 | 24.8 | -5.0 | 0.000 |
| Instructor (186.3) | Associate Professor (401.5) | -215.3 | 23.8 | -9.1 | 0.000 |
| Instructor (186.3) | Full Professor (451.0) | -264.8 | 23.6 | -11.2 | 0.000 |
| Assistant Professor, Teaching (189.6) | Associate Professor, Teaching (194.5) | -4.8 | 45.4 | -0.1 | 1.000 |
| Assistant Professor, Teaching (189.6) | Assistant Professor (308.9) | -119.3 | 31.8 | -3.8 | 0.004 |
| Assistant Professor, Teaching (189.6) | Associate Professor (401.5) | -211.9 | 31.0 | -6.8 | 0.000 |
| Assistant Professor, Teaching (189.6) | Full Professor (451.0) | -261.4 | 30.9 | -8.5 | 0.000 |
| Associate Professor, Teaching (194.5) | Assistant Professor (308.9) | -114.5 | 39.0 | -2.9 | 0.070 |
| Associate Professor, Teaching (194.5) | Associate Professor (401.5) | -207.1 | 38.3 | -5.4 | 0.000 |
| Associate Professor, Teaching (194.5) | Full Professor (451.0) | -256.6 | 38.2 | -6.7 | 0.000 |
| Assistant Professor (308.9) | Associate Professor (401.5) | -92.6 | 20.5 | -4.5 | 0.000 |
| Assistant Professor (308.9) | Full Professor (451.0) | -142.1 | 20.2 | -7.0 | 0.000 |
| Associate Professor (401.5) | Full Professor (451.0) | -49.5 | 19.0 | -2.6 | 0.190 |

- Full professors were awarded a higher amount of Tri-Council grant funding than:
  - Assistant professors
  - Assistant professors, Teaching
  - Associate professors, Teaching
  - Full professors, Teaching
  - Instructors
- Full professors had similar Tri-Council grant funding as Associate Professors
- Associate professors were awarded a higher amount of Tri-Council grant funding than:
  - Assistant professors
  - Assistant professors, Teaching
  - Associate professors, Teaching
  - Full professors, Teaching
  - Instructors
- Assistant professors were awarded a higher amount of Tri-Council grant funding than:
  - Instructors
  - Assistant professors, Teaching
- Assistant professors had similar Tri-Council grant funding as:
  - Associate professors, Teaching
  - Full professors, Teaching
- Tri-Council grant funding did not differ between Professors of Teaching at all ranks (Full, Associate, Assistant) and Instructors

**Physical Therapy faculty: Publication count**

The overall Kruskal-Wallis model for PT publication count was significant:

| **PT publication count** | **Sample size** | **df** | **Test statistic** | **p** |
| --- | --- | --- | --- | --- |
| Appointment title | 262 | 6 | 173.6 | <0.001 |

Post-hoc analysis identified the following differences:

| **Dunn’s pairwise comparison of appointment titles** | | **Mean rank pairwise difference** | **Std. Error** | **Standardized Test Statistic** | **Adj. Sig.** |
| --- | --- | --- | --- | --- | --- |
| **Appointment 1 (Mean rank)** | **Appointment 2 (Mean rank)** |  |  |  |  |
| Instructor (40.3) | Assistant Professor, Teaching (52.8) | -12.5 | 20.6 | -0.6 | 1.000 |
| Instructor (40.3) | Full Professor, Teaching (64.1) | -23.8 | 33.1 | -0.7 | 1.000 |
| Instructor (40.3) | Associate Professor, Teaching (78.5) | -38.2 | 25.7 | -1.5 | 1.000 |
| Instructor (40.3) | Assistant Professor (115.3) | -75.0 | 15.9 | -4.7 | 0.000 |
| Instructor (40.3) | Associate Professor (163.3) | -123.0 | 15.0 | -8.2 | 0.000 |
| Instructor (40.3) | Full Professor (206.4) | -166.1 | 14.8 | -11.2 | 0.000 |
| Assistant Professor, Teaching (52.8) | Full Professor, Teaching (64.1) | -11.3 | 35.3 | -0.3 | 1.000 |
| Assistant Professor, Teaching (52.8) | Associate Professor, Teaching (78.5) | -25.7 | 28.4 | -0.9 | 1.000 |
| Assistant Professor, Teaching (52.8) | Assistant Professor (115.3) | -62.6 | 20.0 | -3.1 | 0.038 |
| Assistant Professor, Teaching (52.8) | Associate Professor (163.3) | -110.5 | 19.4 | -5.7 | 0.000 |
| Assistant Professor, Teaching (52.8) | Full Professor (206.4) | -153.7 | 19.2 | -8.0 | 0.000 |
| Full Professor, Teaching (64.1) | Associate Professor, Teaching (78.5) | 14.4 | 38.5 | 0.4 | 1.000 |
| Full Professor, Teaching (64.1) | Assistant Professor (115.3) | -51.2 | 32.7 | -1.6 | 1.000 |
| Full Professor, Teaching (64.1) | Associate Professor (163.3) | -99.2 | 32.3 | -3.1 | 0.045 |
| Full Professor, Teaching (64.1) | Full Professor (206.4) | -142.3 | 32.2 | -4.4 | 0.000 |
| Associate Professor, Teaching (78.5) | Assistant Professor (115.3) | -36.9 | 25.2 | -1.5 | 1.000 |
| Associate Professor, Teaching (78.5) | Associate Professor (163.3) | -84.8 | 24.7 | -3.4 | 0.013 |
| Associate Professor, Teaching (78.5) | Full Professor (206.4) | -128.0 | 24.6 | -5.2 | 0.000 |
| Assistant Professor (115.3) | Associate Professor (163.3) | -48.0 | 14.3 | -3.4 | 0.017 |
| Assistant Professor (115.3) | Full Professor (206.4) | -91.1 | 14.1 | -6.5 | 0.000 |
| Associate Professor (163.3) | Full Professor (206.4) | -43.1 | 13.1 | -3.3 | 0.022 |

- Full professors had higher publication count than:
  - Assistant professors
  - Associate professors
  - Assistant professors, Teaching
  - Associate professors, Teaching
  - Full professors, Teaching
  - Instructors
- Associate professors had higher publication count than:
  - Assistant professors
  - Assistant professors, Teaching
  - Associate professors, Teaching
  - Full professors, Teaching
  - Instructors
- Assistant professors had higher publication count than
  - Assistant Professors, Teaching
  - Instructors
- Assistant professors had similar publication counts as:
  - Associate professors, Teaching
  - Full professors, Teaching
- Publication count did not differ between Professors of Teaching at all ranks (Full, Associate, Assistant) and Instructors

**Physical Therapy faculty: h-index**

The overall Kruskal-Wallis model for PT h-index was significant:

| **PT h-index** | **Sample size** | **df** | **Test statistic** | **p** |
| --- | --- | --- | --- | --- |
| Appointment title | 262 | 6 | 182.9 | <0.001 |

Post-hoc analysis identified the following differences:

| **Dunn’s pairwise comparison of appointment titles** | | **Mean rank pairwise difference** | **Std. Error** | **Standardized Test Statistic** | **Adj. Sig.** |
| --- | --- | --- | --- | --- | --- |
| **Appointment 1 (Mean rank)** | **Appointment 2 (Mean rank)** |  |  |  |  |
| Instructor (39.7) | Assistant Professor, Teaching (49.4) | -9.7 | 20.6 | -0.5 | 1.000 |
| Instructor (39.7) | Full Professor, Teaching (71.0) | -31.3 | 33.0 | -0.9 | 1.000 |
| Instructor (39.7) | Associate Professor, Teaching (83.3) | -43.5 | 25.6 | -1.7 | 1.000 |
| Instructor (39.7) | Assistant Professor (111.0) | -71.3 | 15.9 | -4.5 | 0.000 |
| Instructor (39.7) | Associate Professor (162.1) | -122.3 | 15.0 | -8.1 | 0.000 |
| Instructor (39.7) | Full Professor (210.6) | -170.9 | 14.8 | -11.5 | 0.000 |
| Assistant Professor, Teaching (49.4) | Full Professor, Teaching (71.0) | -21.6 | 35.2 | -0.6 | 1.000 |
| Assistant Professor, Teaching (49.4) | Associate Professor, Teaching (83.3) | -33.9 | 28.4 | -1.2 | 1.000 |
| Assistant Professor, Teaching (49.4) | Assistant Professor (111.0) | -61.6 | 20.0 | -3.1 | 0.044 |
| Assistant Professor, Teaching (49.4) | Associate Professor (162.1) | -112.6 | 19.4 | -5.8 | 0.000 |
| Assistant Professor, Teaching (49.4) | Full Professor (210.6) | -161.2 | 19.2 | -8.4 | 0.000 |
| Full Professor, Teaching (71.0) | Associate Professor, Teaching (83.3) | 12.3 | 38.4 | 0.3 | 1.000 |
| Full Professor, Teaching (71.0) | Assistant Professor (111.0) | -40.0 | 32.7 | -1.2 | 1.000 |
| Full Professor, Teaching (71.0) | Associate Professor (162.1) | -91.0 | 32.3 | -2.8 | 0.102 |
| Full Professor, Teaching (71.0) | Full Professor (210.6) | -139.6 | 32.2 | -4.3 | 0.000 |
| Associate Professor, Teaching (83.3) | Assistant Professor (111.0) | -27.7 | 25.2 | -1.1 | 1.000 |
| Associate Professor, Teaching (83.3) | Associate Professor (162.1) | -78.8 | 24.7 | -3.2 | 0.030 |
| Associate Professor, Teaching (83.3) | Full Professor (210.6) | -127.4 | 24.6 | -5.2 | 0.000 |
| Assistant Professor (111.0) | Associate Professor (162.1) | -51.0 | 14.3 | -3.6 | 0.007 |
| Assistant Professor (111.0) | Full Professor (210.6) | -99.6 | 14.1 | -7.1 | 0.000 |
| Associate Professor (162.1) | Full Professor (210.6) | -48.6 | 13.1 | -3.7 | 0.005 |

- Full professors had higher h-index than:
  - Assistant professors
  - Associate professors
  - Assistant professors, Teaching
  - Associate professors, Teaching
  - Full professors, Teaching
  - Instructors
- Associate professors had higher h-index than:
  - Assistant professors
  - Assistant professors, Teaching
  - Associate professors, Teaching
  - Instructors
- Associate professors had similar h-index as Full professors, Teaching
- Assistant professors had higher h-index than:
  - Assistant Professors, Teaching
  - Instructors
- Assistant professors had similar h-index as:
  - Associate professors, Teaching
  - Full professors, Teaching
- h-index did not differ between Professors of Teaching at all ranks (Full, Associate, Assistant) and Instructors

**Physical Therapy faculty: Number of Tri-Council grants awarded as PI (CIHR), Project Lead (NSERC), Applicant (SSHRC)**

The overall Kruskal-Wallis model for PT number of Tri-Council grants awarded was significant:

| **PT No. Tri-Council grants** | **Sample size** | **df** | **Test statistic** | **p** |
| --- | --- | --- | --- | --- |
| Appointment title | 262 | 6 | 103.2 | <0.001 |

Post-hoc analysis identified the following differences:

| **Dunn’s pairwise comparison of appointment titles** | | **Mean rank pairwise difference** | **Std. Error** | **Standardized Test Statistic** | **Adj. Sig.** |
| --- | --- | --- | --- | --- | --- |
| **Appointment 1 (Mean rank)** | **Appointment 2 (Mean rank)** |  |  |  |  |
| Instructor (73.0) | Full Professor, Teaching (73.0) | 0.000 | 30.103 | 0.000 | 1.000 |
| Instructor (73.0) | Assistant Professor, Teaching (79.3) | -6.275 | 18.739 | -0.335 | 1.000 |
| Instructor (73.0) | Associate Professor, Teaching (82.7) | -9.682 | 23.362 | -0.414 | 1.000 |
| Instructor (73.0) | Assistant Professor (113.2) | -40.200 | 14.437 | -2.784 | 0.113 |
| Instructor (73.0) | Associate Professor (159.4) | -86.438 | 13.697 | -6.311 | 0.000 |
| Instructor (73.0) | Full Professor (182.5) | -109.464 | 13.499 | -8.109 | 0.000 |
| Full Professor, Teaching (73.0) | Assistant Professor, Teaching (79.3) | 6.275 | 32.106 | 0.195 | 1.000 |
| Full Professor, Teaching (73.0) | Associate Professor, Teaching (82.7) | 9.682 | 35.006 | 0.277 | 1.000 |
| Full Professor, Teaching (73.0) | Assistant Professor (113.2) | -40.200 | 29.801 | -1.349 | 1.000 |
| Full Professor, Teaching (73.0) | Associate Professor (159.4) | -86.438 | 29.450 | -2.935 | 0.070 |
| Full Professor, Teaching (73.0) | Full Professor (182.5) | -109.464 | 29.358 | -3.729 | 0.004 |
| Assistant Professor, Teaching (79.3) | Associate Professor, Teaching (82.7) | -3.407 | 25.892 | -0.132 | 1.000 |
| Assistant Professor, Teaching (79.3) | Assistant Professor (113.2) | -33.925 | 18.249 | -1.859 | 1.000 |
| Assistant Professor, Teaching (79.3) | Associate Professor (159.4) | -80.163 | 17.670 | -4.537 | 0.000 |
| Assistant Professor, Teaching (79.3) | Full Professor (182.5) | -103.189 | 17.517 | -5.891 | 0.000 |
| Associate Professor, Teaching (82.7) | Assistant Professor (113.2) | -30.518 | 22.971 | -1.329 | 1.000 |
| Associate Professor, Teaching (82.7) | Associate Professor (159.4) | -76.756 | 22.513 | -3.409 | 0.014 |
| Associate Professor, Teaching (82.7) | Full Professor (182.5) | -99.782 | 22.393 | -4.456 | 0.000 |
| Assistant Professor (113.2) | Associate Professor (159.4) | -46.238 | 13.019 | -3.552 | 0.008 |
| Assistant Professor (113.2) | Full Professor (182.5) | -69.264 | 12.810 | -5.407 | 0.000 |
| Associate Professor (159.4) | Full Professor (182.5) | -23.026 | 11.970 | -1.924 | 1.000 |

- Full professors were awarded a greater number of Tri-Council grants than:
  - Assistant professors
  - Assistant professors, Teaching
  - Associate professors, Teaching
  - Full professors, Teaching
  - Instructors
- Full professors were awarded a similar number of Tri-Council grants as Associate professors
- Associate professors were awarded a greater number of Tri-Council grants than:
  - Assistant professors
  - Assistant professors, Teaching
  - Associate professors, Teaching
  - Instructors
- Associate professors were awarded a similar number of Tri-Council grants as Full professors, Teaching
- Number of Tri-Council grants awarded did not differ between Assistant professors, Professors of Teaching at all ranks (Full, Associate, Assistant) and Instructors

**Physical Therapy faculty: Tri-Council grant funding awarded as PI (CIHR), Project Lead (NSERC), Applicant (SSHRC)**

The overall Kruskal-Wallis model for PT Tri-Council grant funding awarded was significant:

| **PT Tri-Council grant funding** | **Sample size** | **df** | **Test statistic** | **p** |
| --- | --- | --- | --- | --- |
| Appointment title | 262 | 6 | 100.5 | <0.001 |

Post-hoc analysis identified the following differences:

| **Dunn’s pairwise comparison of appointment titles** | | **Mean rank pairwise difference** | **Std. Error** | **Standardized Test Statistic** | **Adj. Sig.** |
| --- | --- | --- | --- | --- | --- |
| **Appointment 1 (Mean rank)** | **Appointment 2 (Mean rank)** |  |  |  |  |
| Instructor (73.0) | Full Professor, Teaching (73.0) | 0.000 | 30.139 | 0.000 | 1.000 |
| Instructor (73.0) | Assistant Professor, Teaching (79.3) | -6.250 | 18.761 | -0.333 | 1.000 |
| Instructor (73.0) | Associate Professor, Teaching (83.5) | -10.455 | 23.390 | -0.447 | 1.000 |
| Instructor (73.0) | Assistant Professor (115.9) | -42.930 | 14.454 | -2.970 | 0.063 |
| Instructor (73.0) | Associate Professor (156.7) | -83.742 | 13.713 | -6.107 | 0.000 |
| Instructor (73.0) | Full Professor (182.9) | -109.870 | 13.515 | -8.129 | 0.000 |
| Full Professor, Teaching (73.0) | Assistant Professor, Teaching (79.3) | 6.250 | 32.144 | 0.194 | 1.000 |
| Full Professor, Teaching (73.0) | Associate Professor, Teaching (83.5) | 10.455 | 35.048 | 0.298 | 1.000 |
| Full Professor, Teaching (73.0) | Assistant Professor (115.9) | -42.930 | 29.836 | -1.439 | 1.000 |
| Full Professor, Teaching (73.0) | Associate Professor (156.7) | -83.742 | 29.484 | -2.840 | 0.095 |
| Full Professor, Teaching (73.0) | Full Professor (182.9) | -109.870 | 29.392 | -3.738 | 0.004 |
| Assistant Professor, Teaching (79.3) | Associate Professor, Teaching (83.5) | -4.205 | 25.922 | -0.162 | 1.000 |
| Assistant Professor, Teaching (79.3) | Assistant Professor (115.9) | -36.680 | 18.271 | -2.008 | 0.938 |
| Assistant Professor, Teaching (79.3) | Associate Professor (156.7) | -77.492 | 17.691 | -4.380 | 0.000 |
| Assistant Professor, Teaching (79.3) | Full Professor (182.9) | -103.620 | 17.537 | -5.909 | 0.000 |
| Associate Professor, Teaching (83.5) | Assistant Professor (115.9) | -32.475 | 22.998 | -1.412 | 1.000 |
| Associate Professor, Teaching (83.5) | Associate Professor (156.7) | -73.288 | 22.540 | -3.251 | 0.024 |
| Associate Professor, Teaching (83.5) | Full Professor (182.9) | -99.415 | 22.420 | -4.434 | 0.000 |
| Assistant Professor (115.9) | Associate Professor (156.7) | -40.812 | 13.034 | -3.131 | 0.037 |
| Assistant Professor (115.9) | Full Professor (182.9) | -66.940 | 12.825 | -5.219 | 0.000 |
| Associate Professor (156.7) | Full Professor (182.9) | -26.127 | 11.984 | -2.180 | 0.614 |

- Full professors were awarded a higher amount of Tri-Council grant funding than:
  - Assistant professors
  - Assistant professors, Teaching
  - Associate professors, Teaching
  - Full professors, Teaching
  - Instructors
- Full professors were awarded a similar amount of Tri-Council grants as Associate professors
- Associate professors were awarded a higher amount of Tri-Council grant funding as PI than:
  - Assistant professors
  - Assistant professors, Teaching
  - Associate professors, Teaching
  - Instructors
- Associate professors were awarded a similar amount of Tri-Council grant funding as Full professors, Teaching
- Amount of Tri-Council grant funding did not differ between Assistant professors, Professors of Teaching at all ranks (Full, Associate, Assistant) and Instructors

**Occupational Therapy faculty: Publication count**

The overall Kruskal-Wallis model for OT publication count was significant:

| **OT publication count** | **Sample size** | **df** | **Test statistic** | **p** |
| --- | --- | --- | --- | --- |
| Appointment title | 248 | 6 | 129.5 | <0.001 |

Post-hoc analysis identified the following differences:

| **Dunn’s pairwise comparison of appointment titles** | | **Mean rank pairwise difference** | **Std. Error** | **Standardized Test Statistic** | **Adj. Sig.** |
| --- | --- | --- | --- | --- | --- |
| **Appointment 1 (Mean rank)** | **Appointment 2 (Mean rank)** |  |  |  |  |
| Instructor (33.2) | Associate Professor, Teaching (43.4) | -10.3 | 28.8 | -0.4 | 1.000 |
| Instructor (33.2) | Full Professor, Teaching (45.3) | -12.1 | 34.8 | -0.3 | 1.000 |
| Instructor (33.2) | Assistant Professor, Teaching (63.2) | -30.0 | 21.7 | -1.4 | 1.000 |
| Instructor (33.2) | Assistant Professor (106.8) | -73.6 | 16.7 | -4.4 | 0.000 |
| Instructor (33.2) | Associate Professor (154.6) | -121.4 | 16.0 | -7.6 | 0.000 |
| Instructor (33.2) | Full Professor (180.8) | -147.6 | 16.3 | -9.1 | 0.000 |
| Associate Professor, Teaching (43.4) | Full Professor, Teaching (45.3) | -1.8 | 40.9 | -0.0 | 1.000 |
| Associate Professor, Teaching (43.4) | Assistant Professor, Teaching (63.2) | 19.7 | 30.5 | 0.6 | 1.000 |
| Associate Professor, Teaching (43.4) | Assistant Professor (106.8) | -63.3 | 27.1 | -2.3 | 0.412 |
| Associate Professor, Teaching (43.4) | Associate Professor (154.6) | -111.2 | 26.8 | -4.2 | 0.001 |
| Associate Professor, Teaching (43.4) | Full Professor (180.8) | -137.3 | 26.9 | -5.1 | 0.000 |
| Full Professor, Teaching (45.3) | Assistant Professor, Teaching (63.2) | 17.9 | 36.3 | 0.5 | 1.000 |
| Full Professor, Teaching (45.3) | Assistant Professor (106.8) | -61.5 | 33.5 | -1.8 | 1.000 |
| Full Professor, Teaching (45.3) | Associate Professor (154.6) | -109.3 | 33.2 | -3.3 | 0.021 |
| Full Professor, Teaching (45.3) | Full Professor (180.8) | -135.4 | 33.3 | -4.1 | 0.001 |
| Assistant Professor, Teaching (63.2) | Assistant Professor (106.8) | -43.6 | 19.5 | -2.2 | 0.529 |
| Assistant Professor, Teaching (63.2) | Associate Professor (154.6) | -91.4 | 18.9 | -4.8 | 0.000 |
| Assistant Professor, Teaching (63.2) | Full Professor (180.8) | -117.6 | 19.2 | -6.1 | 0.000 |
| Assistant Professor (106.8) | Associate Professor (154.6) | -47.8 | 12.9 | -3.7 | 0.004 |
| Assistant Professor (106.8) | Full Professor (180.8) | -74.0 | 13.2 | -5.6 | 0.000 |
| Associate Professor (154.6) | Full Professor (180.8) | -26.1 | 12.4 | -2.1 | 0.740 |

- Full professors had higher publication count than:
  - Assistant professors
  - Assistant professors, Teaching
  - Associate professors, Teaching
  - Full professors, Teaching
  - Instructors
- Full professors had similar publication count as Associate professors
- Associate professors had higher publication count than:
  - Assistant professors
  - Assistant professors, Teaching
  - Associate professors, Teaching
  - Full professors, Teaching
  - Instructors
- Assistant professors had higher publication count than Instructors
- Assistant professors had similar publication counts as:
  - Assistant Professors, Teaching
  - Associate professors, Teaching
  - Full professors, Teaching
- Publication count did not differ between Professors of Teaching at all ranks (Full, Associate, Assistant) and Instructors

**Occupational Therapy faculty: h-index**

The overall Kruskal-Wallis model for OT h-index was significant:

| **OT h-index** | **Sample size** | **df** | **Test statistic** | **p** |
| --- | --- | --- | --- | --- |
| Appointment title | 248 | 6 | 140.0 | <0.001 |

Post-hoc analysis identified the following differences:

| **Dunn’s pairwise comparison of appointment titles** | | **Mean rank pairwise difference** | **Std. Error** | **Standardized Test Statistic** | **Adj. Sig.** |
| --- | --- | --- | --- | --- | --- |
| **Appointment 1 (Mean rank)** | **Appointment 2 (Mean rank)** |  |  |  |  |
| Instructor (31.9) | Associate Professor, Teaching (46.4) | -14.4 | 28.7 | -0.5 | 1.000 |
| Instructor (31.9) | Full Professor, Teaching (48.1) | -16.2 | 34.8 | -0.5 | 1.000 |
| Instructor (31.9) | Assistant Professor, Teaching (52.5) | -20.6 | 21.7 | -1.0 | 1.000 |
| Instructor (31.9) | Assistant Professor (104.0) | -72.1 | 16.6 | -4.3 | 0.000 |
| Instructor (31.9) | Associate Professor (159.5) | -127.5 | 16.0 | -8.0 | 0.000 |
| Instructor (31.9) | Full Professor (180.7) | -148.7 | 16.3 | -9.1 | 0.000 |
| Associate Professor, Teaching (46.4) | Full Professor, Teaching (48.1) | -1.7 | 40.9 | -0.0 | 1.000 |
| Associate Professor, Teaching (46.4) | Assistant Professor, Teaching (52.5) | 6.1 | 30.5 | 0.2 | 1.000 |
| Associate Professor, Teaching (46.4) | Assistant Professor (104.0) | -57.7 | 27.1 | -2.1 | 0.704 |
| Associate Professor, Teaching (46.4) | Associate Professor (159.5) | -113.1 | 26.7 | -4.2 | 0.000 |
| Associate Professor, Teaching (46.4) | Full Professor (180.7) | -134.2 | 26.9 | -5.0 | 0.000 |
| Full Professor, Teaching (48.1) | Assistant Professor, Teaching (52.5) | 4.4 | 36.2 | 0.1 | 1.000 |
| Full Professor, Teaching (48.1) | Assistant Professor (104.0) | -55.9 | 33.5 | -1.7 | 1.000 |
| Full Professor, Teaching (48.1) | Associate Professor (159.5) | -111.4 | 33.2 | -3.4 | 0.017 |
| Full Professor, Teaching (48.1) | Full Professor (180.7) | -132.6 | 33.3 | -4.0 | 0.001 |
| Assistant Professor, Teaching (52.5) | Assistant Professor (104.0) | -51.5 | 19.5 | -2.6 | 0.170 |
| Assistant Professor, Teaching (52.5) | Associate Professor (159.5) | -107.0 | 18.9 | -5.7 | 0.000 |
| Assistant Professor, Teaching (52.5) | Full Professor (180.7) | -128.2 | 19.2 | -6.7 | 0.000 |
| Assistant Professor (104.0) | Associate Professor (159.5) | -55.4 | 12.9 | -4.3 | 0.000 |
| Assistant Professor (104.0) | Full Professor (180.7) | -76.6 | 13.2 | -5.8 | 0.000 |
| Associate Professor (159.5) | Full Professor (180.7) | -21.2 | 12.4 | -1.7 | 1.000 |

- Full professors had higher h-index than:
  - Assistant professors
  - Assistant professors, Teaching
  - Associate professors, Teaching
  - Full professors, Teaching
  - Instructors
- Full professors had similar h-index as Associate professors
- Associate professors had higher h-index than:
  - Assistant professors
  - Assistant professors, Teaching
  - Associate professors, Teaching
  - Full professors, Teaching
  - Instructors
- Assistant professors had higher h-index than Instructors
- Assistant professors had similar h-index as:
  - Assistant Professors, Teaching
  - Associate professors, Teaching
  - Full professors, Teaching
- h-index did not differ between Professors of Teaching at all ranks (Full, Associate, Assistant) and Instructors

**Occupational Therapy faculty: Number of Tri-Council grants awarded as PI (CIHR), Project Lead (NSERC), Applicant (SSHRC)**

The overall Kruskal-Wallis model for OT number of Tri-Council grants awarded was significant:

| **OT No. Tri-Council grants** | **Sample size** | **df** | **Test statistic** | **p** |
| --- | --- | --- | --- | --- |
| Appointment title | 248 | 6 | 75.0 | <0.001 |

Post-hoc analysis identified the following differences:

| **Dunn’s pairwise comparison of appointment titles** | | **Mean rank pairwise difference** | **Std. Error** | **Standardized Test Statistic** | **Adj. Sig.** |
| --- | --- | --- | --- | --- | --- |
| **Appointment 1 (Mean rank)** | **Appointment 2 (Mean rank)** |  |  |  |  |
| Assistant Professor, Teaching (63.5) | Instructor (66.4) | 2.9 | 20.1 | 0.1 | 1.000 |
| Associate Professor, Teaching (63.5) | Instructor (66.4) | 2.9 | 26.7 | 0.1 | 1.000 |
| Full Professor, Teaching (63.5) | Instructor (66.4) | 2.9 | 32.4 | 0.1 | 1.000 |
| Assistant Professor, Teaching (63.5) | Associate Professor, Teaching (63.5) | 0.0 | 28.3 | 0.0 | 1.000 |
| Assistant Professor, Teaching (63.5) | Full Professor, Teaching (63.5) | 0.0 | 33.7 | 0.0 | 1.000 |
| Assistant Professor, Teaching (63.5) | Assistant Professor (116.0) | -52.5 | 18.1 | -2.9 | 0.078 |
| Assistant Professor, Teaching (63.5) | Associate Professor (151.2) | -87.7 | 17.6 | -5.0 | 0.000 |
| Assistant Professor, Teaching (63.5) | Full Professor (157.6) | -94.1 | 17.8 | -5.3 | 0.000 |
| Associate Professor, Teaching (63.5) | Full Professor, Teaching (63.5) | 0.0 | 38.0 | 0.0 | 1.000 |
| Associate Professor, Teaching (63.5) | Assistant Professor (116.0) | -52.5 | 25.2 | -2.1 | 0.786 |
| Associate Professor, Teaching (63.5) | Associate Professor (151.2) | -87.7 | 24.9 | -3.5 | 0.009 |
| Associate Professor, Teaching (63.5) | Full Professor (157.6) | -94.1 | 25.0 | -3.8 | 0.004 |
| Full Professor, Teaching (63.5) | Assistant Professor (116.0) | -52.5 | 31.2 | -1.7 | 1.000 |
| Full Professor, Teaching (63.5) | Associate Professor (151.2) | -87.7 | 30.9 | -2.8 | 0.094 |
| Full Professor, Teaching (63.5) | Full Professor (157.6) | -94.1 | 31.0 | -3.0 | 0.050 |
| Instructor (66.4) | Assistant Professor (116.0) | -49.6 | 15.5 | -3.2 | 0.028 |
| Instructor (66.4) | Associate Professor (151.2) | -84.8 | 14.9 | -5.7 | 0.000 |
| Instructor (66.4) | Full Professor (157.6) | -91.2 | 15.1 | -6.0 | 0.000 |
| Assistant Professor (116.0) | Associate Professor (151.2) | -35.2 | 12.0 | -2.9 | 0.069 |
| Assistant Professor (116.0) | Full Professor (157.6) | -41.6 | 12.3 | -3.4 | 0.015 |
| Associate Professor (151.2) | Full Professor (157.6) | -6.4 | 11.5 | -0.6 | 1.000 |

- Full professors were awarded a greater number of Tri-Council grants than:
  - Assistant professors
  - Assistant professors, Teaching
  - Associate professors, Teaching
  - Full professors, Teaching
  - Instructors
- Full professors were awarded a similar number of Tri-Council grants as Associate professors
- Associate professors were awarded a greater number of Tri-Council grants than:
  - Assistant professors, Teaching
  - Associate professors, Teaching
  - Instructors
- Associate professors were awarded a similar number of Tri-Council grants as:
  - Assistant professors
  - Full professors, Teaching
- Assistant professors were awarded a greater number of Tri-Council grants Instructors
- Assistant professors were awarded a similar number of Tri-Council grants as:
  - Assistant professors, Teaching
  - Associate professors, Teaching
  - Full professors, Teaching
- Number of Tri-Council grants awarded did not differ between Professors of Teaching at all ranks (Full, Associate, Assistant) and Instructors

**Occupational Therapy faculty: Tri-Council grant funding awarded as PI (CIHR), Project Lead (NSERC), Applicant (SSHRC)**

The overall Kruskal-Wallis model for OT Tri-Council grant funding awarded was significant:

| **OT Tri-Council grant funding** | **Sample size** | **df** | **Test statistic** | **p** |
| --- | --- | --- | --- | --- |
| Appointment title | 248 | 6 | 74.2 | <0.001 |

Post-hoc analysis identified the following differences:

| **Dunn’s pairwise comparison of appointment titles** | | **Mean rank pairwise difference** | **Std. Error** | **Standardized Test Statistic** | **Adj. Sig.** |
| --- | --- | --- | --- | --- | --- |
| **Appointment 1 (Mean rank)** | **Appointment 2 (Mean rank)** |  |  |  |  |
| Assistant Professor, Teaching (63.5) | Instructor (66.0) | 2.4 | 20.2 | 0.1 | 1.000 |
| Associate Professor, Teaching (63.5) | Instructor (66.0) | 2.4 | 26.8 | 0.1 | 1.000 |
| Full Professor, Teaching (63.5) | Instructor (66.0) | 2.4 | 32.5 | 0.1 | 1.000 |
| Assistant Professor, Teaching (63.5) | Associate Professor, Teaching (63.5) | 0.0 | 28.4 | 0.0 | 1.000 |
| Assistant Professor, Teaching (63.5) | Full Professor, Teaching (63.5) | 0.0 | 33.8 | 0.0 | 1.000 |
| Assistant Professor, Teaching (63.5) | Assistant Professor (116.6) | -53.1 | 18.2 | -2.9 | 0.072 |
| Assistant Professor, Teaching (63.5) | Full Professor (153.9) | -90.4 | 17.9 | -5.1 | 0.000 |
| Assistant Professor, Teaching (63.5) | Associate Professor (154.3) | -90.8 | 17.6 | -5.1 | 0.000 |
| Associate Professor, Teaching (63.5) | Full Professor, Teaching (63.5) | 0.0 | 38.1 | 0.0 | 1.000 |
| Associate Professor, Teaching (63.5) | Assistant Professor (116.6) | -53.1 | 25.3 | -2.1 | 0.750 |
| Associate Professor, Teaching (63.5) | Full Professor (153.9) | -90.4 | 25.1 | -3.6 | 0.007 |
| Associate Professor, Teaching (63.5) | Associate Professor (154.3) | -90.8 | 24.9 | -3.6 | 0.006 |
| Full Professor, Teaching (63.5) | Assistant Professor (116.6) | -53.1 | 31.2 | -1.7 | 1.000 |
| Full Professor, Teaching (63.5) | Full Professor (153.9) | -90.4 | 31.1 | -2.9 | 0.076 |
| Full Professor, Teaching (63.5) | Associate Professor (154.3) | -90.8 | 30.9 | -2.9 | 0.070 |
| Instructor (66.0) | Assistant Professor (116.6) | -50.7 | 15.5 | -3.3 | 0.023 |
| Instructor (66.0) | Full Professor (153.9) | -87.9 | 15.2 | -5.8 | 0.000 |
| Instructor (66.0) | Associate Professor (154.3) | -88.3 | 14.9 | -5.9 | 0.000 |
| Assistant Professor (116.6) | Full Professor (153.9) | -37.2 | 12.3 | -3.0 | 0.053 |
| Assistant Professor (116.6) | Associate Professor (154.3) | -37.6 | 12.0 | -3.1 | 0.036 |
| Full Professor (153.9) | Associate Professor (154.3) | 0.4 | 11.6 | 0.0 | 1.000 |

- Full professors were awarded a higher amount of Tri-Council grant funding than:
  - Assistant professors, Teaching
  - Associate professors, Teaching
  - Instructors
- Full professors were awarded a similar amount of Tri-Council grants as:
  - Assistant professors
  - Associate professors
  - Full professors, Teaching
- Associate professors were awarded a higher amount of Tri-Council grant funding than:
  - Assistant professors
  - Assistant professors, Teaching
  - Associate professors, Teaching
  - Instructors
- Associate professors were awarded a similar amount of Tri-Council grant funding as Full professors, Teaching
- Assistant professors were awarded a higher amount of Tri-Council grant funding than Instructors
- Assistant professors were awarded a similar amount of Tri-Council grant funding as Professors of Teaching at all ranks (assistant, associate, full)
- Amount of Tri-Council grant funding did not differ between Professors of Teaching at all ranks (Full, Associate, Assistant) and Instructors

**Communication Sciences and Disorders faculty: Publication count**

The overall Kruskal-Wallis model for CSD publication count was significant:

| **CSD publication count** | **Sample size** | **df** | **Test statistic** | **p** |
| --- | --- | --- | --- | --- |
| Appointment title | 168 | 6 | 106.2 | <0.001 |

Post-hoc analysis identified the following differences:

| **Dunn’s pairwise comparison of appointment titles** | | **Mean rank pairwise difference** | **Std. Error** | **Standardized Test Statistic** | **Adj. Sig.** |
| --- | --- | --- | --- | --- | --- |
| **Appointment 1 (Mean rank)** | **Appointment 2 (Mean rank)** |  |  |  |  |
| Assistant Professor, Teaching (7.7) | Instructor (15.9) | 8.2 | 24.7 | 0.3 | 1.000 |
| Assistant Professor, Teaching (7.7) | Associate Professor, Teaching (22.1) | -14.4 | 28.5 | -0.5 | 1.000 |
| Assistant Professor, Teaching (7.7) | Full Professor, Teaching (47.8) | -40.1 | 40.7 | -1.0 | 1.000 |
| Assistant Professor, Teaching (7.7) | Assistant Professor (64.8) | -57.1 | 23.2 | -2.5 | 0.293 |
| Assistant Professor, Teaching (7.7) | Associate Professor (95.2) | -87.5 | 22.9 | -3.8 | 0.003 |
| Assistant Professor, Teaching (7.7) | Full Professor (124.8) | -117.1 | 22.7 | -5.2 | 0.000 |
| Instructor (15.9) | Associate Professor, Teaching (22.1) | -6.2 | 21.8 | -0.3 | 1.000 |
| Instructor (15.9) | Full Professor, Teaching (47.8) | -31.8 | 36.4 | -0.9 | 1.000 |
| Instructor (15.9) | Assistant Professor (64.8) | -48.9 | 14.3 | -3.4 | 0.013 |
| Instructor (15.9) | Associate Professor (95.2) | -79.3 | 13.9 | -5.7 | 0.000 |
| Instructor (15.9) | Full Professor (124.8) | -108.9 | 13.4 | -8.1 | 0.000 |
| Associate Professor, Teaching (22.1) | Full Professor, Teaching (47.8) | -25.6 | 39.0 | -0.7 | 1.000 |
| Associate Professor, Teaching (22.1) | Assistant Professor (64.8) | -42.6 | 20.1 | -2.1 | 0.711 |
| Associate Professor, Teaching (22.1) | Associate Professor (95.2) | -73.1 | 19.8 | -3.7 | 0.005 |
| Associate Professor, Teaching (22.1) | Full Professor (124.8) | -102.7 | 19.5 | -5.3 | 0.000 |
| Full Professor, Teaching (47.8) | Assistant Professor (64.8) | -17.0 | 35.3 | -0.5 | 1.000 |
| Full Professor, Teaching (47.8) | Associate Professor (95.2) | -47.5 | 35.2 | -1.4 | 1.000 |
| Full Professor, Teaching (47.8) | Full Professor (124.8) | -77.1 | 35.0 | -2.2 | 0.579 |
| Assistant Professor (64.8) | Associate Professor (95.2) | -30.5 | 10.9 | -2.8 | 0.112 |
| Assistant Professor (64.8) | Full Professor (124.8) | -60.1 | 10.4 | -5.8 | 0.000 |
| Associate Professor (95.2) | Full Professor (124.8) | -29.6 | 9.8 | -3.0 | 0.051 |

- Full professors had higher publication count than:
  - Assistant professors
  - Assistant professors, Teaching
  - Associate professors, Teaching
  - Instructors
- Full professors had similar publication counts as:
  - Associate professors
  - Full professors, Teaching
- Associate professors had higher publication count than:
  - Assistant professors, Teaching
  - Associate professors, Teaching
  - Instructors
- Associate professors had similar publication count as:
  - Assistant professors
  - Full professors, Teaching
- Assistant professors had higher publication count than Instructors
- Assistant professors had similar publication counts as:
  - Assistant Professors, Teaching
  - Associate professors, Teaching
  - Full professors, Teaching
- Publication count did not differ between Professors of Teaching at all ranks (Full, Associate, Assistant) and Instructors

**Communication Sciences and Disorders faculty: h-index**

The overall Kruskal-Wallis model for CSD h-index was significant:

| **CSD h-index** | **Sample size** | **df** | **Test statistic** | **p** |
| --- | --- | --- | --- | --- |
| Appointment title | 168 | 6 | 103.0 | <0.001 |

Post-hoc analysis identified the following differences:

| **Dunn’s pairwise comparison of appointment titles** | | **Mean rank pairwise difference** | **Std. Error** | **Standardized Test Statistic** | **Adj. Sig.** |
| --- | --- | --- | --- | --- | --- |
| **Appointment 1 (Mean rank)** | **Appointment 2 (Mean rank)** |  |  |  |  |
| Assistant Professor, Teaching (9.3) | Instructor (15.8) | 6.5 | 24.7 | 0.3 | 1.000 |
| Assistant Professor, Teaching (9.3) | Associate Professor, Teaching (25.5) | -16.2 | 28.5 | -0.6 | 1.000 |
| Assistant Professor, Teaching (9.3) | Full Professor, Teaching (53.0) | -43.7 | 40.7 | -1.1 | 1.000 |
| Assistant Professor, Teaching (9.3) | Assistant Professor (65.9) | -56.5 | 23.2 | -2.4 | 0.311 |
| Assistant Professor, Teaching (9.3) | Associate Professor (93.5) | -84.2 | 22.9 | -3.7 | 0.005 |
| Assistant Professor, Teaching (9.3) | Full Professor (124.8) | -115.5 | 22.7 | -5.1 | 0.000 |
| Instructor (15.8) | Associate Professor, Teaching (25.5) | -9.7 | 21.8 | -0.5 | 1.000 |
| Instructor (15.8) | Full Professor, Teaching (53.0) | -37.2 | 36.3 | -1.0 | 1.000 |
| Instructor (15.8) | Assistant Professor (65.9) | -50.1 | 14.3 | -3.5 | 0.010 |
| Instructor (15.8) | Associate Professor (93.5) | -77.7 | 13.9 | -5.6 | 0.000 |
| Instructor (15.8) | Full Professor (124.8) | -109.0 | 13.4 | -8.1 | 0.000 |
| Associate Professor, Teaching (25.5) | Full Professor, Teaching (53.0) | -27.5 | 39.0 | -0.7 | 1.000 |
| Associate Professor, Teaching (25.5) | Assistant Professor (65.9) | -40.3 | 20.1 | -2.0 | 0.934 |
| Associate Professor, Teaching (25.5) | Associate Professor (93.5) | -68.0 | 19.8 | -3.4 | 0.012 |
| Associate Professor, Teaching (25.5) | Full Professor (124.8) | -99.3 | 19.5 | -5.1 | 0.000 |
| Full Professor, Teaching (53.0) | Assistant Professor (65.9) | -12.8 | 35.3 | -0.4 | 1.000 |
| Full Professor, Teaching (53.0) | Associate Professor (93.5) | -40.5 | 35.1 | -1.2 | 1.000 |
| Full Professor, Teaching (53.0) | Full Professor (124.8) | -71.8 | 35.0 | -2.1 | 0.843 |
| Assistant Professor (65.9) | Associate Professor (93.5) | -27.7 | 10.9 | -2.5 | 0.239 |
| Assistant Professor (65.9) | Full Professor (124.8) | -58.9 | 10.3 | -5.7 | 0.000 |
| Associate Professor (93.5) | Full Professor (124.8) | -31.3 | 9.8 | -3.2 | 0.028 |

- Full professors had higher h-index than:
  - Assistant professors
  - Associate professors
  - Assistant professors, Teaching
  - Associate professors, Teaching
  - Instructors
- Full professors had similar h-index as Full Professors, Teaching
- Associate professors had higher h-index than:
  - Assistant professors, Teaching
  - Associate professors, Teaching
  - Instructors
- Associate professors had similar h-index as:
  - Assistant professors
  - Full professors, Teaching
- Assistant professors had higher h-index than Instructors
- Assistant professors had similar h-index as:
  - Assistant Professors, Teaching Associate professors, Teaching
  - Full professors, Teaching
- h-index did not differ between Professors of Teaching at all ranks (Full, Associate, Assistant) and Instructors

**Communication Sciences and Disorders faculty: Number of Tri-Council grants awarded as PI (CIHR), Project Lead (NSERC), Applicant (SSHRC)**

The overall Kruskal-Wallis model for CSD number of Tri-Council grants awarded was significant:

| **CSD No. Tri-Council grants** | **Sample size** | **df** | **Test statistic** | **p** |
| --- | --- | --- | --- | --- |
| Appointment title | 168 | 6 | 48.5 | <0.001 |

Post-hoc analysis identified the following differences:

| **Dunn’s pairwise comparison of appointment titles** | | **Mean rank pairwise difference** | **Std. Error** | **Standardized Test Statistic** | **Adj. Sig.** |
| --- | --- | --- | --- | --- | --- |
| **Appointment 1 (Mean rank)** | **Appointment 2 (Mean rank)** |  |  |  |  |
| Assistant Professor, Teaching (44.5) | Instructor (49.7) | 5.2 | 22.8 | 0.2 | 1.000 |
| Associate Professor, Teaching (44.5) | Instructor (49.7) | 5.2 | 20.1 | 0.3 | 1.000 |
| Full Professor, Teaching (44.5) | Instructor (49.7) | 5.2 | 33.4 | 0.2 | 1.000 |
| Assistant Professor, Teaching (44.5) | Associate Professor, Teaching (44.5) | 0.0 | 26.2 | 0.0 | 1.000 |
| Assistant Professor, Teaching (44.5) | Full Professor, Teaching (44.5) | 0.0 | 37.4 | 0.0 | 1.000 |
| Assistant Professor, Teaching (44.5) | Assistant Professor (72.7) | -28.2 | 21.3 | -1.3 | 1.000 |
| Assistant Professor, Teaching (44.5) | Associate Professor (82.4) | -37.9 | 21.1 | -1.8 | 1.000 |
| Assistant Professor, Teaching (44.5) | Full Professor (113.8) | -69.2 | 20.9 | -3.3 | 0.019 |
| Associate Professor, Teaching (44.5) | Full Professor, Teaching (44.5) | 0.0 | 35.9 | 0.0 | 1.000 |
| Associate Professor, Teaching (44.5) | Assistant Professor (72.7) | -28.2 | 18.5 | -1.5 | 1.000 |
| Associate Professor, Teaching (44.5) | Associate Professor (82.4) | -37.9 | 18.2 | -2.1 | 0.781 |
| Associate Professor, Teaching (44.5) | Full Professor (113.8) | -69.2 | 17.9 | -3.9 | 0.002 |
| Full Professor, Teaching (44.5) | Assistant Professor (72.7) | -28.2 | 32.5 | -0.9 | 1.000 |
| Full Professor, Teaching (44.5) | Associate Professor (82.4) | -37.9 | 32.3 | -1.2 | 1.000 |
| Full Professor, Teaching (44.5) | Full Professor (113.8) | -69.2 | 32.2 | -2.2 | 0.659 |
| Instructor (49.7) | Assistant Professor (72.7) | -23.1 | 13.2 | -1.8 | 1.000 |
| Instructor (49.7) | Associate Professor (82.4) | -32.7 | 12.8 | -2.6 | 0.217 |
| Instructor (49.7) | Full Professor (113.8) | -64.1 | 12.4 | -5.2 | 0.000 |
| Assistant Professor (72.7) | Associate Professor (82.4) | -9.7 | 10.1 | -1.0 | 1.000 |
| Assistant Professor (72.7) | Full Professor (113.8) | -41.0 | 9.5 | -4.3 | 0.000 |
| Associate Professor (82.4) | Full Professor (113.8) | -31.3 | 9.0 | -3.5 | 0.010 |

- Full professors were awarded a greater number of Tri-Council grants as PI than:
  - Assistant professors
  - Associate professors
  - Assistant professors, Teaching
  - Associate professors, Teaching
  - Instructors
- Full professors were awarded a similar number of Tri-Council grants as Full professors, Teaching
- Number of Tri-Council grants awarded did not differ between Associate Professors, Assistant professors, Professors of Teaching at all ranks (Full, Associate, Assistant) and Instructors

**Communication Sciences and Disorders faculty: Tri-Council grant funding awarded as PI (CIHR), Project Lead (NSERC), Applicant (SSHRC)**

The overall Kruskal-Wallis model for CSD Tri-Council grant funding awarded was significant:

| **CSD Tri-Council grant funding** | **Sample size** | **df** | **Test statistic** | **p** |
| --- | --- | --- | --- | --- |
| Appointment title | 168 | 6 | 45.6 | <0.001 |

Post-hoc analysis identified the following differences:

| **Dunn’s pairwise comparison of appointment titles** | | **Mean rank pairwise difference** | **Std. Error** | **Standardized Test Statistic** | **Adj. Sig.** |
| --- | --- | --- | --- | --- | --- |
| **Appointment 1 (Mean rank)** | **Appointment 2 (Mean rank)** |  |  |  |  |
| Assistant Professor, Teaching (44.5) | Instructor (49.1) | 4.6 | 22.9 | 0.2 | 1.000 |
| Associate Professor, Teaching (44.5) | Instructor (49.1) | 4.6 | 20.2 | 0.2 | 1.000 |
| Full Professor, Teaching (44.5) | Instructor (49.1) | 4.6 | 33.6 | 0.1 | 1.000 |
| Assistant Professor, Teaching (44.5) | Associate Professor, Teaching (44.5) | 0.0 | 26.4 | 0.0 | 1.000 |
| Assistant Professor, Teaching (44.5) | Full Professor, Teaching (44.5) | 0.0 | 37.7 | 0.0 | 1.000 |
| Assistant Professor, Teaching (44.5) | Assistant Professor (73.1) | -28.6 | 21.5 | -1.3 | 1.000 |
| Assistant Professor, Teaching (44.5) | Associate Professor (84.3) | -39.8 | 21.2 | -1.9 | 1.000 |
| Assistant Professor, Teaching (44.5) | Full Professor (112.3) | -67.8 | 21.0 | -3.2 | 0.026 |
| Associate Professor, Teaching (44.5) | Full Professor, Teaching (44.5) | 0.0 | 36.1 | 0.0 | 1.000 |
| Associate Professor, Teaching (44.5) | Assistant Professor (73.1) | -28.6 | 18.6 | -1.5 | 1.000 |
| Associate Professor, Teaching (44.5) | Associate Professor (84.3) | -39.8 | 18.3 | -2.2 | 0.626 |
| Associate Professor, Teaching (44.5) | Full Professor (112.3) | -67.8 | 18.0 | -3.8 | 0.004 |
| Full Professor, Teaching (44.5) | Assistant Professor (73.1) | -28.6 | 32.7 | -0.9 | 1.000 |
| Full Professor, Teaching (44.5) | Associate Professor (84.3) | -39.8 | 32.5 | -1.2 | 1.000 |
| Full Professor, Teaching (44.5) | Full Professor (112.3) | -67.8 | 32.4 | -2.1 | 0.763 |
| Instructor (49.1) | Assistant Professor (73.1) | -24.0 | 13.2 | -1.8 | 1.000 |
| Instructor (49.1) | Associate Professor (84.3) | -35.2 | 12.9 | -2.7 | 0.128 |
| Instructor (49.1) | Full Professor (112.3) | -63.2 | 12.4 | -5.1 | 0.000 |
| Assistant Professor (73.1) | Associate Professor (84.3) | -11.2 | 10.1 | -1.1 | 1.000 |
| Assistant Professor (73.1) | Full Professor (112.3) | -39.2 | 9.6 | -4.1 | 0.001 |
| Associate Professor (84.3) | Full Professor (112.3) | -28.0 | 9.0 | -3.1 | 0.041 |

- Full professors were awarded a higher amount of Tri-Council grant funding than:
  - Assistant professors
  - Associate Professors
  - Assistant professors, Teaching
  - Associate professors, Teaching
  - Instructors
- Full professors were awarded a similar amount of Tri-Council grants as Full professors, Teaching
- Amount of Tri-Council grant funding did not differ between Associate professors, Assistant professors, Professors of Teaching at all ranks (Full, Associate, Assistant) and Instructors
