# Supplementary material for: Research productivity and impact of rehabilitation science faculty across Canada: An observational study of publicly available and survey data
Source: PLoS One. 2026 Sep 15;21(9):e0357806. doi: 10.1371/journal.pone.0357806 (PMC13577373; doi:10.1371/journal.pone.0357806)
Supplement: Supplementary file 6 [file pone.0357806.s006.docx]

**Supplement 6 – Regression models**

Table 1. General linear model examining associations with h-index for the total population of rehabilitation science faculty across Canada (n=685).

| Model (R^2^ = 0.93) | **β (95% CI)** | **p-value** | **η²p** | **Interpretation** |
| --- | --- | --- | --- | --- |
| Intercept | 0.366 (0.233 to 0.499) | <0.001 | 0.042 | - |
| Publication count | 0.624 (0.597 to 0.650) | <0.001 | 0.763 | 1% increase in publications associated with 0.62% higher h-index (95%CI 0.60 to 0.65) |
| Sum of Tri-Council grant funding awarded | 0.002 (-0.002 to 0.007) | 0.323 | 0.001 | Tri-Council grant funding awarded is not significantly associated with h-index |
| Discipline  RS  CSD  OT  PT | 0.085 (-0.139 to 0.309)  0.002 (-0.058 to 0.061)  -0.022 (-0.074 to 0.030)  Reference | Overall: 0.664  0.455  0.955  0.410  - | 0.002  0.001  <0.001  0.001  - | Discipline is not significantly associated with h-index |
| Appointment title  Instructor  Asst Prof, Teaching  Assoc Prof, Teaching  Full Prof, Teaching  Asst Prof  Assoc Prof  Full Prof | -0.300 (-0.416 to -0.183)  -0.339 (-0.464 to -0.214)  -0.128 (-0.268 to 0.011)  -0.191 (-0.372 to -0.010)  -0.155 (-0.228 to -0.082)  -0.077 (-0.139 to -0.015)  Reference | Overall: <0.001  <.001  <.001  0.072  0.038  <.001  0.015  - | 0.052  0.037  0.041  0.005  0.006  0.025  0.009  - | Compared with Full Prof:  26% lower h-index (95%CI -34 to -17%)  29% lower h-index (95%CI -37 to -19%)  Not significantly associated  17% lower h-index (95%CI -31 to -1%)  14% lower h-index (95%CI -20 to -8%)  7% lower h-index (95%CI -13 to -1%) |

- All continuous variables are natural log +1 transformed; log-log model reported. Parameter estimates with robust standard errors (heteroskedasticity-consistent, HC3) are reported.
- Continuous variable coefficients can be interpreted as a 1% increase in x predictor variable leads to an estimated β% change in h-index, holding other variables constant. For example, a 1% increase in publication count is associated with an estimated 0.62% increase in h-index (95% CI: 0.60% to 0.65%).
- Categorical variable coefficients can be interpreted as percent difference compared to the reference group using the equation (*e*^β^ -1) x 100, holding other variables constant. For example, Instructors had an estimated e^-0.3^ -1 x 100 = 26% lower h-index (95%CI: -34% to -17%) compared with Full Professors).
- Sum of Tri-Council grant funding awarded reflects the sum of funding as PI (CIHR), Project Lead (NSERC) and Applicant (SSHRC).
- Asst=Assistant; Assoc=Associate; CI=Confidence Interval; CSD=Communication Sciences and Disorders; OT=Occupational Therapy; Prof=Professor; PT=Physical Therapy; RS=Rehabilitation Scientist.

Table 2. Mixed effects model examining associations with h-index for the total population of rehabilitation science faculty across Canada (n=685), accounting for academic unit clustering.

| Model: R^2^ fixed effects = 0.93; R^2^ fixed + random effects = 0.94 | | | |
| --- | --- | --- | --- |
| **Fixed effects** | **β (95% CI)** | **p-value** | **Interpretation** |
| Intercept | 0.456 (0.308 to 0.604) | <0.001 | - |
| Publication count | 0.610 (0.584 to 0.636) | <0.001 | 1% increase in publications associated with 0.61% higher h-index (95%CI 0.58 to 0.64) |
| Sum of Tri-Council grant funding awarded | 0.002 (-0.002 to 0.006) | 0.39 | Tri-Council grant funding awarded is not significantly associated with h-index |
| Discipline  RS  CSD  OT  PT | 0.074 (-0.202 to 0.351)  -0.035 (-0.157 to 0.087)  -0.040 (-0.154 to 0.074)  Reference | Overall: 0.771  0.594  0.561  0.475  - | Discipline is not significantly associated with h-index |
| Appointment title  Instructor  Asst Prof, Teaching  Assoc Prof, Teaching  Full Prof, Teaching  Asst Prof  Assoc Prof  Full Prof | -0.413 (-0.529 to -0.298)  -0.420 (-0.550 to -0.289)  -0.244 (-0.381 to 0.107)  -0.208 (-0.381 to -0.036)  -0.209 (-0.282 to -0.136)  -0.106 (-0.167 to -0.045)  Reference | Overall: <0.001  <0.001  <0.001  <0.001  0.018  <0.001  <0.001  - | Compared with Full Prof:  34% lower h-index (95%CI -41 to -26%)  34% lower h-index (95%CI -42 to -25%)  22% lower h-index (95%CI -32 to -10%)  19% lower h-index (95%CI -32 to -4%)  19% lower h-index (95%CI -25 to -13%)  10% lower h-index (95%CI -15 to -4%) |
| **Random effect** | **τ₀₀ variance (95% CI)** | **p-value** |  |
| Academic unit  ICC=0.186 | 0.017 (0.009 to 0.034) | 0.003 | Of variance in h-index unexplained by fixed effects, 18.6% is explained by academic unit |

- All continuous variables are natural log +1 transformed; log-log model reported.
- Continuous variable coefficients can be interpreted as a 1% increase in x predictor variable leads to an estimated β% change in h-index, holding other variables constant. For example, a 1% increase in publication count is associated with an estimated 0.61% increase in h-index (95% CI: 0.58% to 0.64%).
- Categorical variable coefficients can be interpreted as percent difference compared to the reference group using the equation (*e*^β^ -1) x 100, holding other variables constant. For example, Instructors had an estimated e^-0.413^ -1 x 100 = 34% lower h-index (95%CI: -41% to -26%) compared with Full Professors).

Table 3. Mixed effects model examining associations with h-index for the subset of rehabilitation science faculty across Canada with complete research environment data contributed by unit Heads (n=401 faculty across 22 academic units), accounting for academic unit clustering.

| Model: R^2^ fixed effects = 0.95; R^2^ fixed + random effects = 0.95 | | | |
| --- | --- | --- | --- |
| **Fixed effects** | **β (95% CI)** | **p-value** | **Interpretation** |
| Intercept | 0.215 (-0.462 to 0.892) | 0.508 | - |
| Publication count | 0.634 (0.601 to 0.668) | <0.001 | 1% increase in publications associated with 0.63% higher h-index (95%CI 0.60 to 0.67) |
| Sum of Tri-Council grant funding awarded | 0.000 (-0.005 to 0.006) | 0.854 | Tri-Council grant funding awarded is not significantly associated with h-index |
| Discipline  CSD  OT  PT | 0.121 (-0.032 to 0.274)  0.042 (-0.091 to 0.174)  Reference | Overall: 0.278  0.114  0.511  - | Discipline is not significantly associated with h-index |
| Appointment title  Instructor  Asst Prof, Teaching  Assoc Prof, Teaching  Full Prof, Teaching  Asst Prof  Assoc Prof  Full Prof | -0.437 (-0.584 to -0.291)  -0.494 (-0.667 to -0.320)  -0.211 (-0.382 to -0.039)  -0.249 (-0.472 to -0.026)  -0.257 (-0.350 to -0.164)  -0.174 (-0.253 to -0.096)  Reference | Overall: <0.001  <0.001  <0.001  0.016  0.029  <0.001  <0.001  - | Compared with Full Prof:  35% lower h-index (95%CI -44 to -25%)  39% lower h-index (95%CI -49 to -27%)  19% lower h-index (95%CI -32 to -4%)  22% lower h-index (95%CI -38 to -3%)  23% lower h-index (95%CI -30 to -15%)  16% lower h-index (95%CI -22 to -9%) |
| Unit-level research supports | <-0.001 (-0.259 to 0.259) | 1.0 | Unit-level research supports is not significantly associated with h-index |
| Unit-level research funding received in past 5 years | 0.008 (-0.033 to 0.049) | 0.695 | Unit level research funding received in past 5 years is not significantly associated with h-index |
| Unit-level number of trainees | 0.017 (-0.074 to 0.107) | 0.705 | Unit level number of trainees is not significantly associated with h-index |
| **Random effect** | **τ₀₀ variance (95% CI)** | **p-value** |  |
| Academic unit  ICC=0.110 | 0.0008 (0.003 to 0.025) | 0.071 | Of variance in h-index unexplained by fixed effects, 11.0% is explained by academic unit |

- All continuous variables are natural log +1 transformed; log-log model reported
- Continuous variable coefficients can be interpreted as a 1% increase in x predictor variable leads to an estimated β% change in h-index, holding other variables constant. For example, a 1% increase in publication count is associated with an estimated 0.63% increase in h-index (95% CI: 0.60% to 0.67%).
- Categorical variable coefficients can be interpreted as percent difference compared to the reference group using the equation (*e*^β^ -1) x 100, holding other variables constant. For example, Instructors had an estimated e^-0.437^ -1 x 100 = 35% lower h-index (95%CI: -44% to -25%) compared with Full Professors).

Table 4. General linear model examining associations with sum of Tri-Council grant funding awarded for the total population of rehabilitation science faculty across Canada (n=685).

| Model (R^2^ = 0.41) | **B (95% CI)** | **p-value** | **η²p** | **Interpretation** |
| --- | --- | --- | --- | --- |
| Intercept | -0.710 (-3.052 to 1.631) | 0.552 | 0.001 | - |
| Publication count | 1.770 (0.848 to 2.691) | <0.001 | 0.021 | 1% increase in publications associated with 1.77% increase in Tri-Council grant funding (95%CI 0.85 to 2.69) |
| h-index | 0.656 (-0.647 to 1.959) | 0.323 | 0.001 | h-index is not significantly associated with Tri-Council grant funding |
| Discipline  RS  CSD  OT  PT | 1.984 (-1.875 to 5.843)  -0.200 (-1.224 to 0.824)  0.416 (-0.483 to 1.315)  Reference | Overall: 0.471  0.313  0.701  0.364  - | 0.004  0.002  0.000  0.001  - | Discipline is not significantly associated with Tri-Council grant funding |
| Appointment title  Instructor  Asst Prof, Teaching  Assoc Prof, Teaching  Full Prof, Teaching  Asst Prof  Assoc Prof  Full Prof | -1.883 (-3.923 to 0.158)  -3.060 (-5.244 to -0.876)  -3.532 (-5.934 to -1.131)  -4.245 (-7.355 to -1.136)  -1.751 (-3.020 to -0.482)  -0.514 (-1.587 to 0.559)  Reference | Overall: 0.010  0.070  0.006  0.004  0.008  0.007  0.347  - | 0.025  0.005  0.011  0.012  0.011  0.011  0.001  - | Compared with Full Prof:  Not significantly associated  95% lower funding (95%CI -99 to -58%)  97% lower funding (95%CI -100 to -68%)  99% lower funding (95%CI -100 to -68%)  83% lower funding (95%CI -95 to -38%)  Not significantly associated |

- All continuous variables are natural log +1 transformed; log-log model reported. Parameter estimates with robust standard errors (heteroskedasticity-consistent, HC3) are reported.
- Continuous variable coefficients can be interpreted as a 1% increase in x predictor variable leads to an estimated β% change in Tri-Council grant funding awarded, holding other variables constant. For example, a 1% increase in publication count is associated with an estimated 1.77% increase in Tri-Council grant funding awarded (95% CI: 0.85% to 2.69%).
- Categorical variable coefficients can be interpreted as percent difference compared to the reference group using the equation (*e*^β^ -1) x 100, holding other variables constant. For example, Assistant Professors had an estimated e^-1.751^ -1 x 100 = 83% lower Council grant funding awarded (95%CI: -95 to -38%) Tri- compared with Full Professors).
- Sum of Tri-Council grant funding awarded reflects the sum of funding as PI (CIHR), Project Lead

Table 5. Mixed effects model examining associations with sum of Tri-Council funding awarded for the total population of rehabilitation science faculty across Canada (n=685), accounting for academic unit clustering.

| Model (R^2^ = 0.416 for fixed effects only, R^2^ = 0.425 for fixed and random effects) | | | |
| --- | --- | --- | --- |
| **Fixed effects** | **β (95% CI)** | **p-value** | **Interpretation** |
| Intercept | 0.680 (-3.073 to 1.712) | 0.576 | - |
| Publication count | 1.754 (0.827 to 2.682) | <0.001 | 1% increase in publications associated with 1.75% higher grant funding (95%CI 0.83 to 2.68) |
| h-index | 0.659 (-0.657 to 1.975 | 0.326 | h-index is not significantly associated with Tri-Council grant funding awarded |
| Discipline  RS  CSD  OT  PT | 1.976 (-1.967 to 5.919)  -0.147 (-1.340 to 1.046)  0.419 (-0.651 to 1.490)  Reference | Overall: 0.58  0.325  0.804  0.429  - | Discipline is not significantly associated with Tri-Council grant funding awarded |
| Appointment title  Instructor / Lecturer  Asst Prof, Teaching  Assoc Prof, Teaching  Full Prof, Teaching  Asst Prof  Assoc Prof  Full Prof | -1.902 (-3.968 to 0.165)  -3.238 (-5.485 to -0.990)  -3.615 (-6.035 to -1.195)  -4.213 (-7.322 to -1.104)  -1.717 (-3.003 to -0.432)  -0.475 (-1.556 to 0.606)  Reference | Overall: 0.009  0.071  0.005  0.003  0.008  0.009  0.389  - | Compared with Full Prof:  Not significantly associated  96% lower funding (95%CI -0.5 to -100%)  97% lower funding (95%CI -0.3 to -100%)  99% lower funding (95%CI -0.8 to -100%)  82% lower funding (95%CI -0.9 to -95%)  Not significantly associated |
| **Random effect** | **τ₀₀ variance (95% CI)** | **p-value** |  |
| Academic unit  ICC: 0.016 | 0.429 (0.044 to 4.211) | 0.391 | Of variance in grant funding unexplained by fixed effects, 1.6% is explained by academic unit |

- All continuous variables are natural log +1 transformed; log-log model reported.
- Sum of Tri-Council grant funding awarded reflects the sum of funding as PI (CIHR), Project Lead (NSERC) and Applicant (SSHRC).
- Continuous variable coefficients can be interpreted as a 1% increase in x predictor variable leads to an estimated β% change in h-index, holding other variables constant. For example, a 1% increase in publication count is associated with an estimated 1.75% increase in Tri-Council grant funding received (95% CI: 0.83% to 2.68%).
- Categorical variable coefficients can be interpreted as percent difference compared to the reference group using the equation (*e*^β^ -1) x 100, holding other variables constant. For example, Assistant Professors had an estimated e^-1.717^ -1 x 100 = 82% lower Tri-Council grant funds received (95%CI: -0.5% to -95%) compared with Full Professors).
